# Performance analysis of a three-dimensional micromixer with baffles using a flexible physics-informed neural network

**DOI:** 10.1038/s41598-026-40254-7

**Published:** 2026-02-21

**Authors:** Meraj Hassanzadeh, Ehsan Ghaderi, Mohamad Ali Bijarchi

**Affiliations:** Department of Mechanical Engineering, Sharif University of Technology, Tehran, Iran

**Keywords:** Micromixer, Microfluidics, Physics-informed neural network (PINN), Transfer learning, Adaptive loss weighting, Computational fluid dynamics (CFD), Engineering, Mathematics and computing, Physics

## Abstract

This study introduces a Flexible Physics-Informed Neural Network (FlexPINN) to overcome the limitations of standard PINNs in modeling fully three-dimensional, geometrically complex micromixers with internal baffles. The mesh-free framework integrates parallel-network architecture, first-order dimensionless governing equations, adaptive loss weighting, and a novel global conservation penalty to prevent trivial solutions and ensure robust convergence. Employing transfer learning reduces the training time for new baffle shapes by ~ 35%, requiring approximately 3.5 h versus 5.5 h for a base case on a single GPU. Validated against conventional Computational Fluid Dynamics, FlexPINN achieves high-fidelity predictions, with maximum relative errors of 3.25% for the pressure drop coefficient and 2.86% for the mixing index. A comprehensive parametric study evaluates three baffle shapes (rectangular, elliptical, triangular) across four configurations and Reynolds numbers (Re = 5, 20, 40, 80). Results demonstrate that rectangular baffles in a double-unit, staggered configuration (C) at Re = 40 yield the peak mixing efficiency of 1.63, significantly outperforming other designs. This work successfully bridges a critical gap in PINN applications by providing a validated, efficient tool for the analysis and optimization of intricate 3D passive micromixers.

## Introduction

Fluid mixing has consistently garnered significant attention in microfluidics research due to its critical role in enhancing the performance of microscale systems. This process is fundamental to a wide range of applications, including chemical synthesis, biomedical diagnostics, pharmaceutical analysis, and lab-on-a-chip systems^1,2^. A key challenge in micromixer design lies in the trade-off between mixing efficiency and pressure drop, enhancing one often compromises the other^3,4^. Generally, fluid mixing methods are categorized into two primary approaches: active techniques, which rely on external energy sources such as electric, magnetic, thermal, or acoustic fields to stimulate and improve mixing performance^5–11^; and passive techniques, which eliminate the need for external power by leveraging innovative microchannel design, increased interfacial contact between fluids, or the integration of structures like porous media and internal baffles^12–15^.

Numerous studies have demonstrated that passive mixing can be significantly enhanced through deliberate geometric modifications. Investigations have ranged from 2D baffle designs analyzed with finite element methods^16^ to the fabrication and testing of innovative 2D passive mixers showing substantial improvement over baseline designs^17^. In three dimensions, research has focused on optimizing obstacle parameters like height, geometry, and number^18^, as well as employing sampling techniques like Latin Hypercube Sampling (LHS) for multi-objective optimization of geometric ratios^19^. These and other works underscore the critical influence of geometric configuration on passive micromixer performance^20–23^. Extensive research using conventional CFD has firmly established the effectiveness of integrating baffles and three-dimensional features. Foundational work has compared the performance of 2D and 3D baffled structures, with 3D geometries introducing transverse flow rotation and achieving over 80% greater mixing efficiency than a standard T-mixer at low Reynolds numbers^24^. Subsequent studies have systematically dissected the impact of baffle shape, identifying rectangular and trapezoidal designs as generating stronger, more stable flow perturbations than triangular ones^25^, and have demonstrated the simultaneous enhancement of mixing and heat transfer using triangular baffles in millimeter-scale T-mixers^26^. The exploration of fully three-dimensional chaotic geometries, such as serpentine and grooved micromixers, has further advanced the understanding of mixing mechanisms in complex 3D flows^27^. Within more constrained 3D T-type geometries, research has progressed to optimizing the layout^28^ and even the flexible, variable-angle arrangement^29^ of internal obstacles. Other innovative designs have combined channel curvature with radial baffles to generate multidirectional vortices, significantly boosting mixing performance^30^. Collectively, this body of literature provides a comprehensive performance benchmark and confirms that geometric complexity is a primary driver of mixing enhancement.

Conventional numerical simulations, such as CFD, rely on spatial/temporal discretization of the governing equations. For complex 3D geometries like those with intricate baffles, this approach becomes computationally expensive and time-consuming due to the challenges of mesh generation and the sensitivity of solutions to mesh quality^20,31,32^. This limits their practicality for rapid parametric studies and design optimization. In recent years, deep learning has emerged as a complementary tool. While data-driven neural networks can infer flow parameters from simulation or experimental data^33–36^, their dependence on large, high-fidelity datasets limits applicability^37,38^. Physics-Informed Neural Networks (PINNs) offer a paradigm shift by directly embedding the governing physical equations into the learning process^39^. This allows PINNs to solve forward and inverse problems with sparse or even no labeled data, leveraging the physics-based loss as a regularizer^40,41^. Consequently, PINNs have seen rapid adoption across fluid mechanics^42,43^, heat transfer^44–46^, and biofluid dynamics^47,48^. Their mesh-free nature presents a potential advantage for simulating complex geometries where traditional meshing is a bottleneck. In microfluidics, PINNs have shown promise in modeling specific phenomena. Applications include predicting multi-species transport in 2D domains^49^, validating against FEM for coupled electrokinetic flows^50^, and solving the Young–Laplace equation for capillary action^51^. However, these studies primarily address simpler 2D physics or specific, lower-dimensional aspects of 3D systems. Focusing specifically on micromixing in microfluidic systems, Chang et al.^52^ investigated an electro-osmotic active micromixer, using PINNs to predict solute transport and electric potential distributions. Although their model performed well overall, significant errors were observed in velocity predictions near electrode regions. This highlights both the promise and current limitations of PINNs in regions with strong local gradients. In summary, above mentioned literature highlights the advantages of the proposed approach and presents a promising future for PINN-based methods in multiphysics predictions for other microfluidic devices. A review of previous studies shows that so far, no research has been conducted on the use of the PINN method in 3D micromixers. On the other hand, due to the inherently three-dimensional nature of the mixing phenomenon, a 3D analysis is essential. However, because solving 3D problems using the PINN method is quite challenging, only a few studies, such as those by Zhang and Zhao^53^ and Biswas and Anand^54^ have investigated simple flow in straight channels without the presence of baffles and solving mass transfer equation.

In this study, the continuity, momentum, and species transport equations are solved using an innovative PINN framework, termed FlexPINN, developed to address the challenges of simulating fluid mixing in three-dimensional microfluidic microchannels featuring internal baffles. The considered straight rectangular microchannels incorporate arrays of corner-mounted baffles with various shapes, including rectangular, triangular, and elliptical geometries, which introduce strong three-dimensional flow complexity and nonlinear species transport behavior. Despite extensive studies on passive micromixers, the design space of such channels remains largely unexplored when the precise sequencing and corner-wise permutation of multiple baffles within a single mixing unit are treated as high-dimensional design variables. With four possible corner locations in a rectangular cross-section, the number of distinct configuration permutations grows rapidly, yet the systematic influence of these spatial sequences on the trade-off between mixing index and pressure drop has not been established. To enable efficient exploration of this complex design space, FlexPINN extends the conventional PINN architecture through a combination of parallel and series neural network structures, reformulated governing equations based on dimensionless first-order derivatives, and adaptive learning strategies to enhance convergence. In addition, transfer learning is employed for network weight initialization across different flow conditions, significantly reducing computational cost. This framework enables an investigation of Reynolds number, baffle shape, baffle configuration, and baffle unit sequencing, and their coupled effects on pressure drop coefficient, mixing index, and mixing efficiency, thereby providing a robust and computationally efficient tool for deriving generalizable design principles for high-performance straight-channel passive micromixers.

## Problem description

### Geometry

**Fig. 1:**
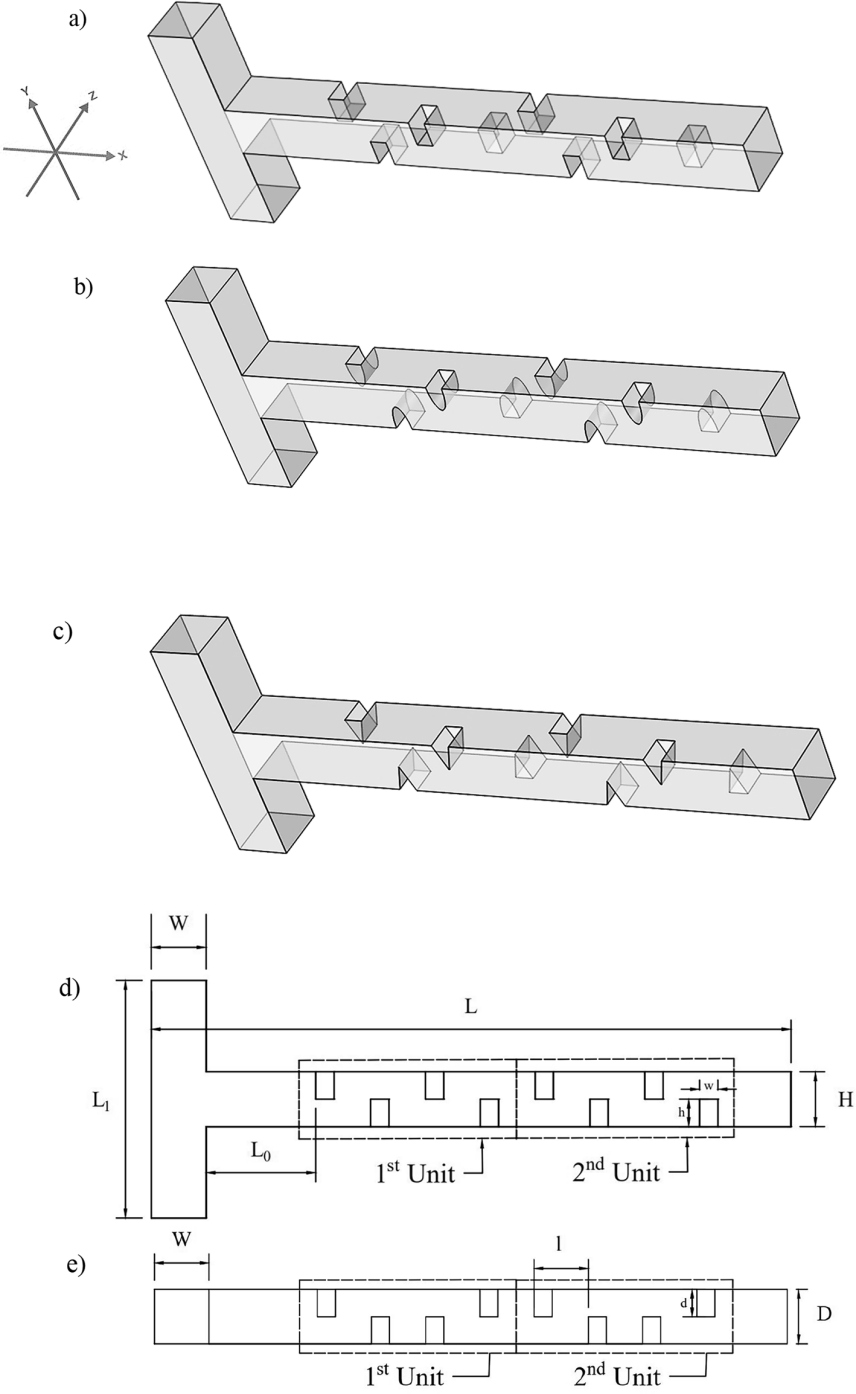
Schematic of the problem for baffle geometry: (**a**) rectangular baffles, (**b**) elliptical baffles, (**c**) triangular baffles, (**d**) microchannel dimensions with baffles in the $xy$ plane, and (**e**) microchannel dimensions with baffles in the $xz$-plane.

This study investigates the fluid flow within a T-shaped micromixer with varying baffle geometries and configurations. A schematic of the problem geometry, along with its dimensions, is shown in Fig. 1. Three different types of baffles (rectangular, elliptical, and triangular) are considered in this research. The baffle shape is designed such that, for all three cases, the height (h) of the baffles is identical. Similarly, the width (w) of the baffles for all three shapes are kept constant. Additionally, the study examines the microchannel with both a single unit (4 baffles) and two units (8 baffles), as illustrated in the figure. Table 1 presents the dimensions and specifications of the three-dimensional microchannel, including the baffles. The selected dimensions (e.g., channel width/height of 300 µm, baffle features ≥ 100 µm) are consistent with standard microfabrication scales, ensuring the designs are practically manufacturable for experimental validation^11,30^.

**Table 1:** Geometric specifications of the microchannel including baffles.

Notation	Definition	Value
L (1 Unit)	The Length of the single-unit microchannel	2.4 mm
L (2 Units)	The Length of the double-unit microchannel	3.5 mm
L0	First baffle inlet distance	0.6 mm
L1	The length of the inlet of the microchannel	1.3 mm
D	The depth of the microchannel	0.3 mm
H	The height of the microchannel	0.3 mm
W	The width of the microchannel inlet	0.3 mm
d	The depth of the baffle	0.15 mm
h	The height of the baffle	0.15 mm
w	The width of the baffle	0.1 mm
l	Distance between two consecutive baffles	0.3 mm

**Fig. 2:**
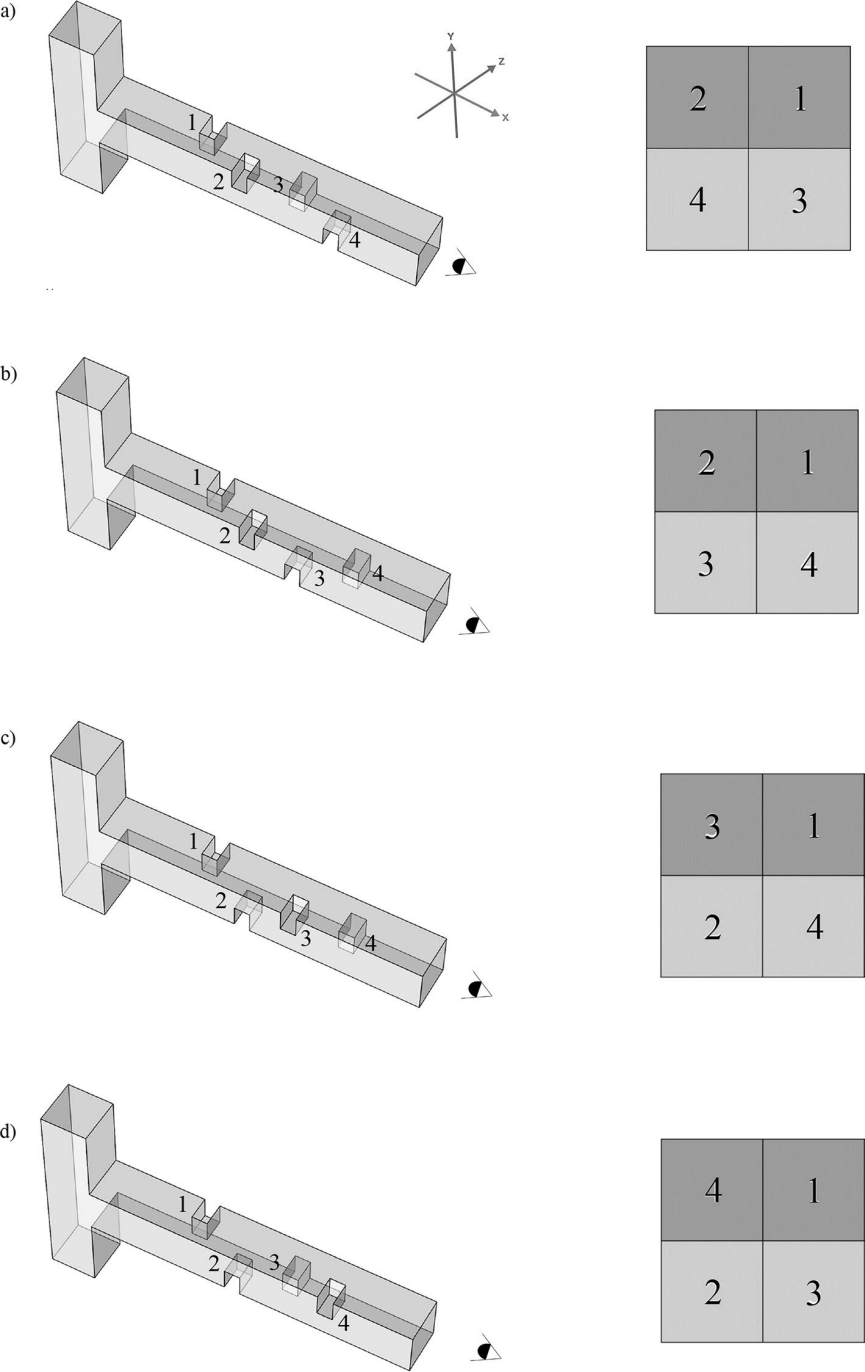
Different baffle configuration in the microchannel with a single unit: (**a**) configuration A, (**b**) configuration B, (**c**) configuration C, and (**d**) configuration D.

Given that this study is a three-dimensional investigation, the different baffle configurations within the microchannel are also considered. Figure 2 illustrates the four baffle configurations examined for a single-unit microchannel. These configurations are referred to as Configurations A, B, C, and D. In each configuration, the baffles are numbered according to the sequence in which the fluid flow encounters them. The images on the left in this figure represent the 3D views, while those on the right illustrate the placement of the baffles within the main channel.

### Governing equations and boundary conditions

Assuming the continuum condition is satisfied in the dimensions of the micromixer, the governing differential equations of the problem in steady-state three dimensional, are derived according to Eqs. (1–3)^55^.

1 $$\nabla .\overrightarrow{U}=0$$

2 $$\rho (\overrightarrow{U}.\nabla )\overrightarrow{U}= -\nabla p+\mu {\nabla }^{2}\overrightarrow{U}$$

3 $$\left(\overrightarrow{U}.\nabla \right)\mathrm{c}={\mathrm{D}}_{\mathrm{c}}{\nabla }^{2}\mathrm{c}$$

These equations, which include the continuity, momentum, and mass transfer equations, includes first and second order of special derivatives. To expedite the solution process, these equations are written in the form of first-order derivatives^56^, which ensures that the backward differentiation process (Backpropagation) occurs only once in the neural network. Hence, they are expressed in terms of lower-order and dimensionless derivatives in this study, according to Eqs. (4–17) in the Cartesian coordinate. The right-hand side of the subsequent equations has been assigned the residual value instead of zero, as the objective within the methodology section is to drive these residual values towards zero. Moreover, to increase solution accuracy, the dimensionless form of the equations is used^48^, which balances the terms in the loss function. Equation (4) represents the continuity equation, Eqs. (5–7) represent the momentum equations in three directions, and Eq. (14) represents the mass transfer equation. Other equations are constitutive equations that assist in the conservation equations. In these relations, $\overrightarrow{U}$ is the velocity vector, ${u}^{*}$, ${v}^{*}$, and ${w}^{*}$ are the dimensionless velocity components in the $x$,$y$, and $z$ directions, correspondingly. Additionally, ${p}^{*}$, ${\tau }^{*}$, and ${c}^{*}$ represent the dimensionless pressure, dimensionless stress, and dimensionless concentration, respectively. Moreover, $\rho$, $\mu$, ${D}_{c}$, and ${U}_{m}$ are the density, viscosity, diffusion coefficient of concentration, and the mean velocity of the fluid in the main channel, respectively. In these relations, ${J}^{*}$ is the dimensionless concentration flux, and the Reynolds (Re) and Schmidt (Sc) numbers are defined in Eqs. (18) and (19)^55^. Re quantifies the ratio of inertial forces to viscous forces within a fluid flow, determining its regime from laminar to turbulent. Sc is the ratio of momentum diffusivity (viscosity) to mass diffusivity, characterizing the relative effectiveness of momentum and mass transport in a fluid medium^64^.

4 $$\frac{\partial {u}^{*}}{\partial {x}^{*}}+\frac{\partial {v}^{*}}{\partial {y}^{*}}+\frac{\partial {w}^{*}}{\partial {z}^{*}}={r}_{GE,1}$$

5 $$\left({u}^{*}\frac{\partial {u}^{*}}{\partial {x}^{*}}+{v}^{*}\frac{\partial {u}^{*}}{\partial {y}^{*}}+{w}^{*}\frac{\partial {u}^{*}}{\partial {z}^{*}}\right)-\frac{\partial {\tau }_{xx}^{*}}{\partial {x}^{*}}- \frac{\partial {\tau }_{xy}^{*}}{\partial {x}^{*}}- \frac{\partial {\tau }_{xz}^{*}}{\partial {x}^{*}}={r}_{GE,2}$$

6 $$\left({u}^{*}\frac{\partial {v}^{*}}{\partial {x}^{*}}+{v}^{*}\frac{\partial {v}^{*}}{\partial {y}^{*}}+{w}^{*}\frac{\partial {v}^{*}}{\partial {z}^{*}}\right)- \frac{\partial {\tau }_{xy}^{*}}{\partial {y}^{*}} - \frac{\partial {\tau }_{yy}^{*}}{\partial {y}^{*}}- \frac{\partial {\tau }_{yz}^{*}}{\partial {y}^{*}}={r}_{GE,3}$$

7 $$\left({u}^{*}\frac{\partial {w}^{*}}{\partial {x}^{*}}+{v}^{*}\frac{\partial {w}^{*}}{\partial {y}^{*}}+{w}^{*}\frac{\partial {w}^{*}}{\partial {z}^{*}}\right)- \frac{\partial {\tau }_{xz}^{*}}{\partial {z}^{*}} - \frac{\partial {\tau }_{yz}^{*}}{\partial {z}^{*}}- \frac{\partial {\tau }_{zz}^{*}}{\partial {z}^{*}}={r}_{GE,4}$$

8 $$-{p}^{*} +\frac{2}{\mathrm{Re}}\frac{\partial {u}^{*}}{\partial {x}^{*}} - {\tau }_{xx}^{*}={r}_{GE,5}$$

9 $$-{p}^{*} +\frac{2}{\mathrm{Re}}\frac{\partial {v}^{*}}{\partial {y}^{*}} - {\tau }_{yy}^{*}={r}_{GE,6}$$

10 $$-{p}^{*} +\frac{2}{\mathrm{Re}}\frac{\partial {w}^{*}}{\partial {z}^{*}} - {\tau }_{zz}^{*}={r}_{GE,7}$$

11 $$\frac{1}{\mathrm{Re}} \left(\frac{\partial {u}^{*}}{\partial {y}^{*}}+\frac{\partial {v}^{*}}{\partial {x}^{*}}\right)- {\tau }_{xy}^{*}={r}_{GE,8}$$

12 $$\frac{1}{\mathrm{Re}} \left(\frac{\partial {u}^{*}}{\partial {z}^{*}}+\frac{\partial {w}^{*}}{\partial {x}^{*}}\right)- {\tau }_{xz}^{*}={r}_{GE,9}$$

13 $$\frac{1}{\mathrm{Re}} \left(\frac{\partial {v}^{*}}{\partial {z}^{*}}+\frac{\partial {w}^{*}}{\partial {y}^{*}}\right)- {\tau }_{yz}^{*}={r}_{GE,10}$$

14 $$\left({u}^{*}\frac{\partial {c}^{*}}{\partial {x}^{*}}+{v}^{*}\frac{\partial {c}^{*}}{\partial {y}^{*}}+{w}^{*}\frac{\partial {c}^{*}}{\partial {z}^{*}}\right)+ \frac{1}{\mathrm{ReSc}}\left(\frac{\partial {J}_{x}^{*}}{\partial {x}^{*}} +\frac{\partial {J}_{y}^{*}}{\partial {y}^{*}} +\frac{\partial {J}_{z}^{*}}{\partial {z}^{*}}\right)={r}_{GE,11}$$

15 $${J}_{x}^{*}+ \frac{\partial {c}^{*}}{\partial {x}^{*}}={r}_{GE,12}$$

16 $${J}_{y}^{*}+ \frac{\partial {c}^{*}}{\partial {y}^{*}}={r}_{GE,13}$$

17 $${J}_{z}^{*}+ \frac{\partial {c}^{*}}{\partial {z}^{*}}={r}_{GE,14}$$

18 $$\mathrm{Re}=\frac{\rho {U}_{mean}D}{\mu }$$

19 $$\mathrm{Sc}=\frac{\mu }{\rho {D}_{c}}$$

**Fig. 3:**
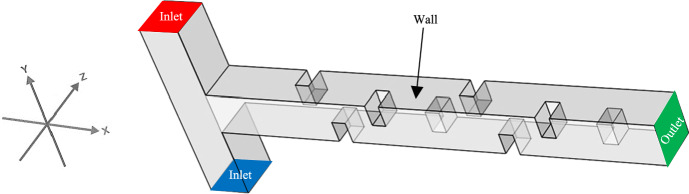
The boundary conditions of the problem. Blue and red represent the first and second inlets, respectively; gray denotes the channel walls, and green indicates the outlet.

The boundary conditions of the problem are illustrated in Fig. 3. The boundary conditions are given in Eqs. (20–35), where Eqs. (20–27) correspond to the inlet boundary conditions, Eqs. (28–29) correspond to the outlet boundary conditions, and Eqs. (30–35) represent the wall boundary conditions. Parabolic velocity profiles are considered at both inlets, with the concentration boundary condition set to unity at the top and zero at the bottom of the T-shape. A parabolic velocity profile is imposed at the inlets of the T-shaped microchannel, consistent with the classical assumption of hydrodynamically fully developed laminar flow in the straight channel segments upstream of the junction. This approach represents a standard and physically validated boundary condition for the simulation of such micromixer configurations, as established in prior literature^11,18^. Additionally, at the outlet of the microchannel, the concentration flux and pressure are set to zero, while on the walls of the microchannel and baffles, no-slip boundary condition is applied (all velocity components and concentration flux are set to zero).

20 $${u}^{*}\left({x}^{*}=\left[0,\frac{{W}^{*}}{2}\right],{y}^{*}=\frac{{L}_{1}^{*}+{D}^{*}}{2},{z}^{*}=\left[0,\frac{{H}^{*}}{2}\right]\right)={r}_{BC,1}$$

21 $${u}^{*}\left({x}^{*}=\left[0,\frac{{W}^{*}}{2}\right],{y}^{*}=\frac{-{L}_{1}^{*}}{2},{z}^{*}=\left[0,\frac{{H}^{*}}{2}\right]\right)={r}_{BC,2}$$

22 $${v}^{*}\left({x}^{*}=\left[0,\frac{{W}^{*}}{2}\right],{y}^{*}=\frac{{L}_{1}^{*}+{D}^{*}}{2},{z}^{*}=\left[0,\frac{{H}^{*}}{2}\right]\right)+\left(\frac{16}{{{D}^{*}}^{4}} \right)\left({z}^{*}{D}^{*} - {z}^{*2}\right)\left({x}^{*}{D}^{*} -{x}^{*2}\right)={r}_{BC,3}$$

23 $${v}^{*}\left({x}^{*}=\left[0,\frac{{W}^{*}}{2}\right],{y}^{*}=\frac{-{L}_{1}^{*}}{2},{z}^{*}=\left[0,\frac{{H}^{*}}{2}\right]\right)-\left(\frac{16}{{{D}^{*}}^{4}} \right)\left({z}^{*}{D}^{*} - {z}^{*2}\right)\left({x}^{*}{D}^{*} -{x}^{*2}\right)={r}_{BC,4}$$

24 $${w}^{*}\left({x}^{*}=\left[0,\frac{{W}^{*}}{2}\right],{y}^{*}=\frac{{L}_{1}^{*}+{D}^{*}}{2},{z}^{*}=\left[0,\frac{{H}^{*}}{2}\right]\right)={r}_{BC,5}$$

25 $${w}^{*}\left({x}^{*}=\left[0,\frac{{W}^{*}}{2}\right],{y}^{*}=\frac{-{L}_{1}^{*}}{2},{z}^{*}=\left[0,\frac{{H}^{*}}{2}\right]\right)={r}_{BC,6}$$

26 $${c}^{*}\left({x}^{*}=\left[0,\frac{{W}^{*}}{2}\right],{y}^{*}=\frac{{L}_{1}^{*}+{D}^{*}}{2},{z}^{*}=\left[0,\frac{{H}^{*}}{2}\right]\right)- 1={r}_{BC,7}$$

27 $${c}^{*}\left({x}^{*}=\left[0,\frac{{W}^{*}}{2}\right],{y}^{*}=\frac{-{L}_{1}^{*}}{2},{z}^{*}=\left[0,\frac{{H}^{*}}{2}\right]\right)= {r}_{BC,8}$$

28 $${p}^{*}\left({x}^{*}={L}^{*},{y}^{*}=[0,{D}^{*}],{z}^{*}=[0,{W}^{*}]\right)= {r}_{BC,9}$$

29 $${J}_{x}^{*}\left({x}^{*}={L}^{*},{y}^{*}=[0,{D}^{*}],{z}^{*}=[0,{W}^{*}]\right)= {r}_{BC,10}$$

30 $${u}^{*}\left({x}_{w}^{*},{y}_{w}^{*},{z}_{w}^{*}\right)= {r}_{BC,11}$$

31 $${v}^{*}\left({x}_{w}^{*},{y}_{w}^{*},{z}_{w}^{*}\right)= {r}_{BC,12}$$

32 $${w}^{*}\left({x}_{w}^{*},{y}_{w}^{*},{z}_{w}^{*}\right)= {r}_{BC,13}$$

33 $${J}_{x}^{*}\left({x}_{w}^{*},{y}_{w}^{*},{z}_{w}^{*}\right) . {n}_{{x}^{*}}= {r}_{BC,14}$$

34 $${J}_{y}^{*}\left({x}_{w}^{*},{y}_{w}^{*},{z}_{w}^{*}\right) . {n}_{{y}^{*}}= {r}_{BC,15}$$

35 $${J}_{z}^{*}\left({x}_{w}^{*},{y}_{w}^{*},{z}_{w}^{*}\right) . {n}_{{z}^{*}}= {r}_{BC,16}$$

### Solution methodology

**Fig. 4:**
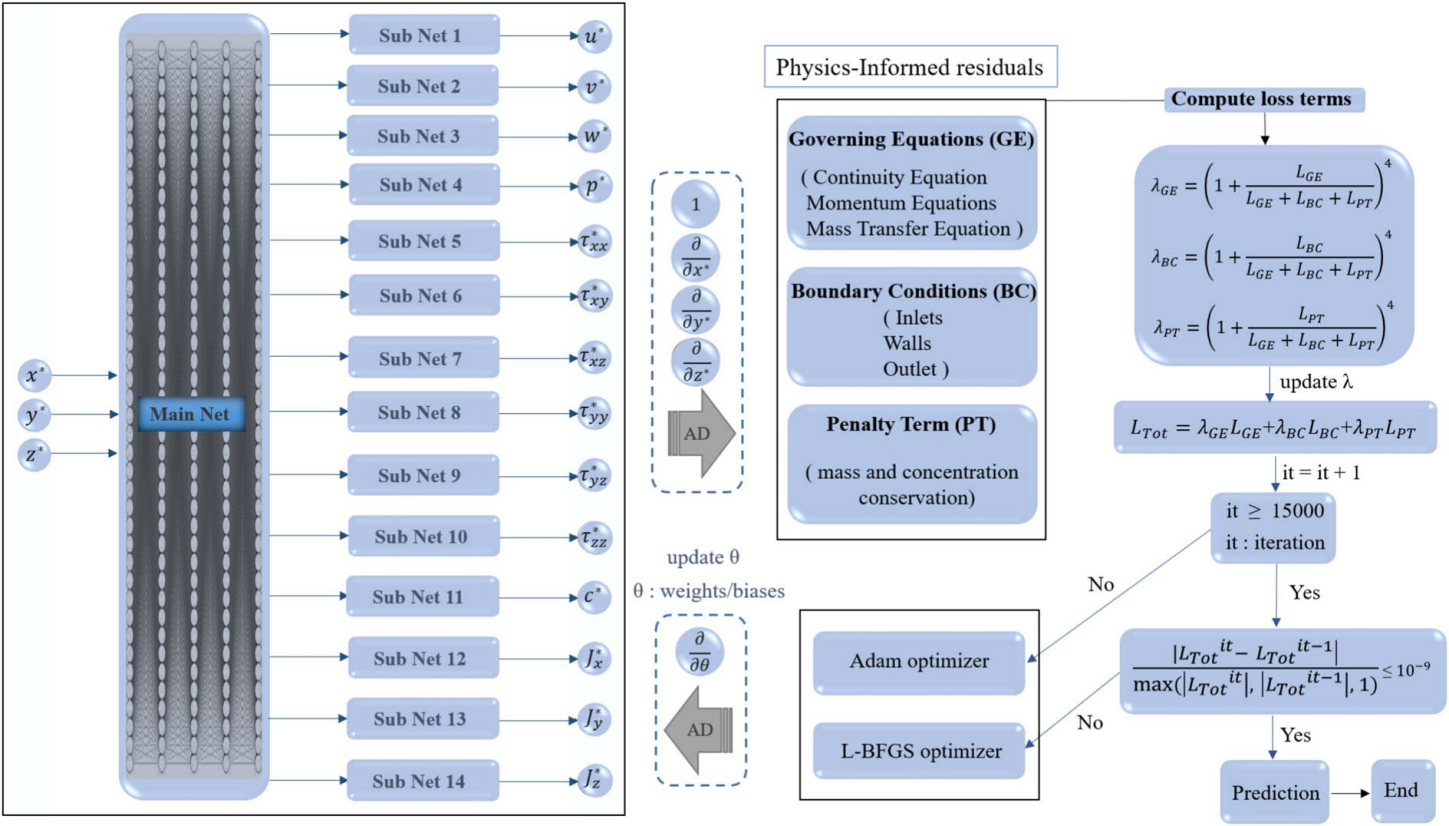
Architecture of the enhanced Physics-Informed Neural Network (FlexPINN) employed in this study. Spatial coordinates $({x}^{*},{y}^{*},{z}^{*})$ are input into a main network with parallel subnetworks to predict 14 output quantities. The total loss ${L}_{total}={L}_{GE}+{L}_{BC}+{L}_{PT}$​ enforces governing equations, boundary conditions, and penalty term (see Eqs. 36–38 for definitions). Hyperbolic tangent (tanh) activation functions and a hybrid Adam/L-BFGS optimization strategy are used.

In the present study, an enhanced PINN approach is employed to solve the governing equations, which include the continuity equation, momentum equations in the $x$, $y$, and $z$ directions, and the mass transfer equation, as defined in Eqs. (4–17). A schematic of the network architecture is presented in Fig. 4. The input to the network consists of the three spatial coordinates ($x$, $y$, $z$), which are fed into 14 parallel sub networks responsible for predicting the 14 desired output quantities. Furthermore, the hyperbolic tangent activation function has been employed in the layers of the neural network. As mentioned above, to accelerate the solution process, the equations are reformulated in terms of first-order derivatives. This approach enables the backpropagation to compute derivatives only once, significantly reducing the computational cost. Furthermore, to maintain a balanced loss function and enhance the stability of the learning process, the non-dimensionalized form of the governing equations with first-order derivatives is used as the loss term of the network. Given the three-dimensional complexity of the problem and the associated challenges of applying conventional PINN methods in such settings, mass flux and bulk concentration constraints at selected channel cross-sections are introduced as penalty terms in the loss function. To enforce global conservation and prevent the trivial zero-velocity solution, a novel penalty term ${L}_{PT}$​ is incorporated into the total loss function. This term ensures that the bulk concentration and the average flow rate remain consistent between the inlet and user-defined control cross-sections along the channel. The bulk average concentration ${c}_{mean}^{*}$ at a given cross-sectional plane *S* is calculated by integrating the predicted concentration field ${c}^{*}(x)$ over that plane: ${c}_{mean}^{*}=\frac{1}{{A}_{S}}{\int }_{S}{c}^{*}\hspace{0.17em}dA$, where ${A}_{S}$ is the area of plane *S*. Similarly, the average dimensionless velocity ${U}_{mean}^{*}$ is computed as: ${{U}^{*}}_{mean}=\frac{1}{{A}_{S}}{\int }_{S}{u}^{*}\hspace{0.17em}dA$, where ${u}^{*}$ is the streamwise velocity component. In practice, these integrals are approximated via summation over the collocation points. Prior studies have recommended the inclusion of such penalty terms in problems with complex geometries or physics^57^.

It should be noted that the loss function employed in Fig. 4 can be defined according to Eq. 36.

36 $${\mathcal{L}}_{GE}=\frac{1}{{N}_{\mathrm{GE}}}\sum_{i=1}^{14}\sum_{j=1}^{{N}_{\mathrm{GE}}}{r}_{\mathrm{GE},i}^{2}, {\mathcal{L}}_{GE}=\sum_{i=1}^{16}\frac{1}{{N}_{\mathrm{BC},i}}\sum_{j=1}^{{N}_{\mathrm{BC},i}}{r}_{\mathrm{BC},i}^{2}, {\mathcal{L}}_{PT}=\frac{1}{{N}_{PT}}\sum_{i=1}^{2}\sum_{j=1}^{{N}_{PT}}{r}_{PT,i}^{2}$$

In the above equation, ${\mathrm{r}}_{PT}$ (the residual Penalty Term) signifies that the mass flow rate and the mean concentration remain constant across different channel section, in accordance with Eq. 37 and 38, respectively^57^. In Eq. 37, the mass flow rate remains constant across different sections. Additionally, in Eq. 38, the cross-sectional area of the channel is denoted by *A*. In Eq. 38, the term 0.5 represents the average concentration between the two inlets, which should remain constant along the channel.

37 $${{U}^{*}}_{mean}-{{U}^{*}}_{inlet}={\mathrm{r}}_{PT,1}$$

38 $${c}_{mean}^{*}-0.5={\mathrm{r}}_{PT,2}$$

Additionally, the loss function weights are adaptively updated during training to further improve convergence. To accelerate the learning process, transfer learning is implemented: the network trained on rectangular baffle shape is initialized using Xavier initialization^58^, and the final parameters of this network are then used to initialize networks for elliptical and triangular baffle shapes. The proposed framework is termed “Flexible” (FlexPINN) to denote its capacity to handle variations within a defined class of three-dimensional microfluidic geometries, specifically, straight T-mixers with internal baffles of differing shapes (rectangular, elliptical, triangular) and configurations. The flexibility is achieved through architectural choices (parallel networks), training strategies (transfer learning), and penalty terms that stabilize learning for this specific physics (steady, incompressible laminar flow). The success of transfer learning is contingent upon geometric and physical similarity. Therefore, the demonstrated flexibility is bounded within the context of parametric variations of baffled straight-channel mixers, which is a prevalent and practically relevant design space in passive microfluidics. A hybrid optimization strategy combining the Adam^59^ and L-BFGS^60^ optimizers has been adopted to train the proposed neural network and optimize its unknown parameters. Further details regarding this implementation are provided in the results section. In the figure, the term ${L}_{GE}$ represents the loss function associated with the governing equations, the term ${L}_{BC}$ corresponds to the loss function related to the boundary conditions, and the term ${L}_{PT}$ denotes the penalty terms. Additionally, the neural network parameters, including weights and biases, which constitute the unknowns of the network, are denoted by the symbol $\theta$ in the figure. In this architecture, the main network is a fully connected neural network (FNN) with 8 hidden layers of 81 neurons each. The parallel subnetworks each consist of 3 layers with 10 neurons.

**Fig. 5:**
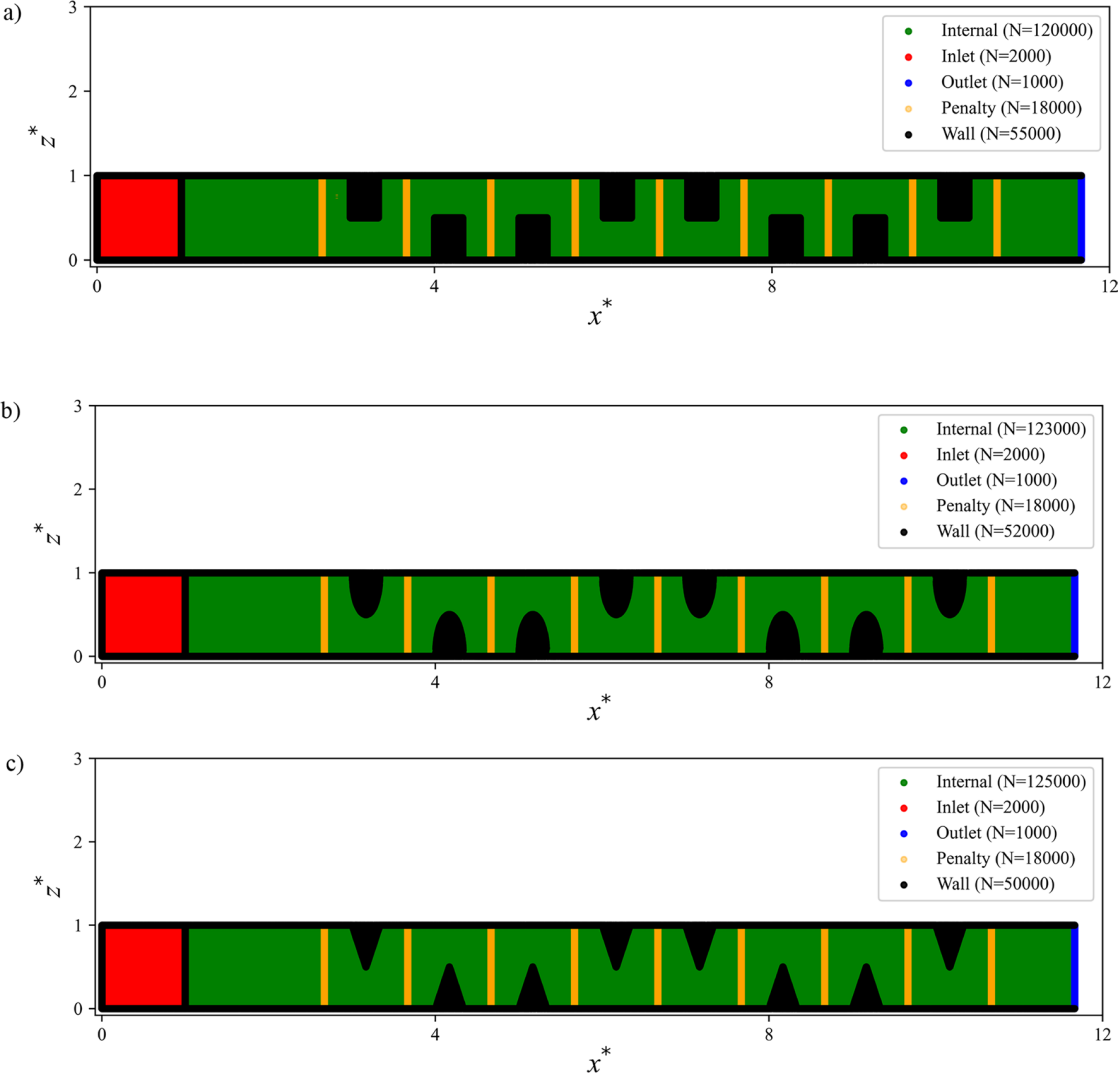
Point distributions generated using Latin Hypercube Sampling (LHS) for three different baffle shapes, shown on an $xz$-plane at mid-height of the main channel: (**a**) rectangular baffles, (**b**) elliptical baffles, and (**c**) triangular baffles.

To simulate the problem using the proposed method, random points were distributed using Latin Hypercube Sampling (LHS)^61^ throughout the computational domain and along the boundaries of the geometry. Figure 5 shows the point distribution and their count on a representative $xz$-plane located at the mid-height of the main channel. For further details on the point distribution and their configuration across other planes, please refer to the GitHub repository associated with this study.

## Results and discussion

In this study, the FlexPINN method—whose network structure is illustrated in Fig. 4—was employed to simulate the 3D fluid flow and mass transfer phenomena in a microchannel equipped with baffles. The simulations were conducted for single-unit and double-unit microchannel, four different baffle placement configurations, three baffle shapes, and four Reynolds numbers (5, 20, 40, and 80) at a constant Schmidt number of 1000. The results presented in this study validate the FlexPINN framework for the analysis of passive mixing in the laminar flow regime (Re ≤ 80), which is the dominant operational regime for many practical microfluidic applications due to manageable pressure drops. At low Reynolds numbers (below ~ 100) where the flow remains laminar, mixing between two fluids in a straight microchannel is challenging due to the absence of turbulence, as molecular diffusion becomes dominant and decelerates the mixing mechanism. To address this, various baffle configurations were employed to enhance mixing. Given the presence of baffles within the microchannel, several previous studies have analyzed the flow behavior under laminar conditions within the Reynolds number range considered in this work^62^.

### Convergence history

**Fig. 6:**
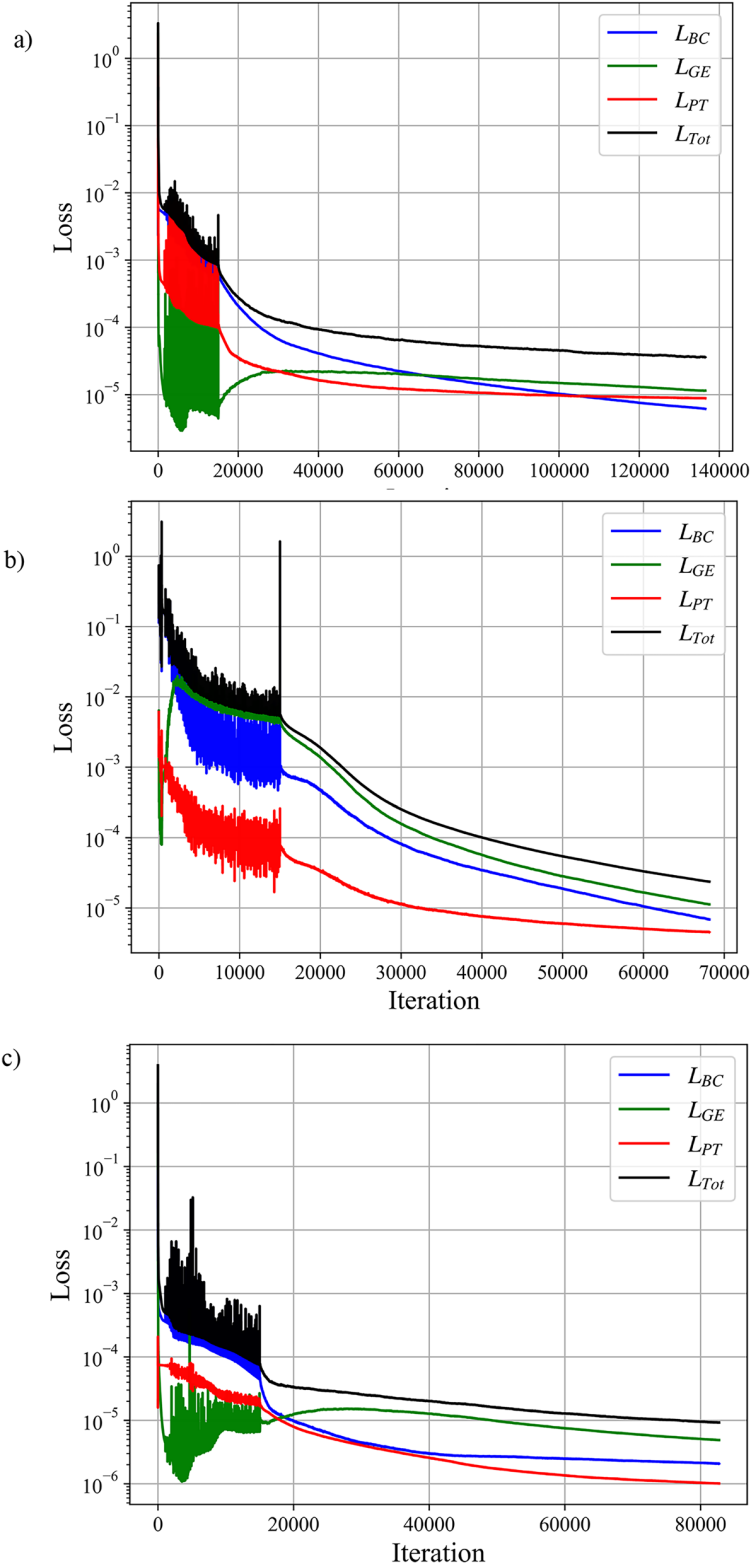
Loss function curves for boundary conditions (${L}_{BC}$), governing equations (${L}_{GE}$), penalty terms (${L}_{PT}$), and total loss (${L}_{Tot}$): (**a**) rectangular baffles, (**b**) elliptical baffles, and (**c**) triangular baffles for Re = 5, Sc = 1000, double-unit microchannel, and configuration A.

Figure 6 illustrates the variation of the loss function terms versus iterations for the rectangular, elliptical, and triangular baffle shapes, respectively in a double-unit microchannel at Re = 5. The sudden change in the loss curves at iteration 15,000 corresponds to the switch from the ADAM optimizer to L-BFGS, implemented to improve solution accuracy. As shown, convergence is achieved around 70,000 and 80,000 iterations for the elliptical and triangular baffles, respectively, while the rectangular baffle requires approximately 140,000 iterations. The accelerated convergence observed for the elliptical and triangular cases is attributed to the use of transfer learning in FlexPINN, resulting in a 35% reduction in training time. The implementation was carried out in Python using the PyTorch library, which supports Automatic Differentiation (AD)^63^. Simulations were performed on an RTX 4080 GPU with 16 GB RAM, requiring approximately 5.5 h for the rectangular case and 3.5 h for the elliptical and triangular baffles with transfer learning. For context, a high-fidelity CFD simulation of an equivalent case (including mesh generation and solving) required approximately 2.5 h on a computing cluster using 8 CPU cores. This demonstrates that the proposed FlexPINN framework is computationally feasible for solving this challenging class of three-dimensional problems. The current computational investment represents a foundational step; ongoing advances in PINN architectures, optimizer efficiency, and GPU hardware are expected to reduce these costs significantly. More importantly, this work establishes that a mesh-free, physics-informed approach can successfully resolve the complex flow fields and mixing dynamics in intricate 3D baffled geometries, which is a prerequisite for its future application in rapid parametric screening and design optimization where its workflow advantages would be fully realized. It is worth noting that a vanilla PINN fails to solve the complex double-unit baffle geometry addressed in this study.

### Grid independency

**Fig. 7:**
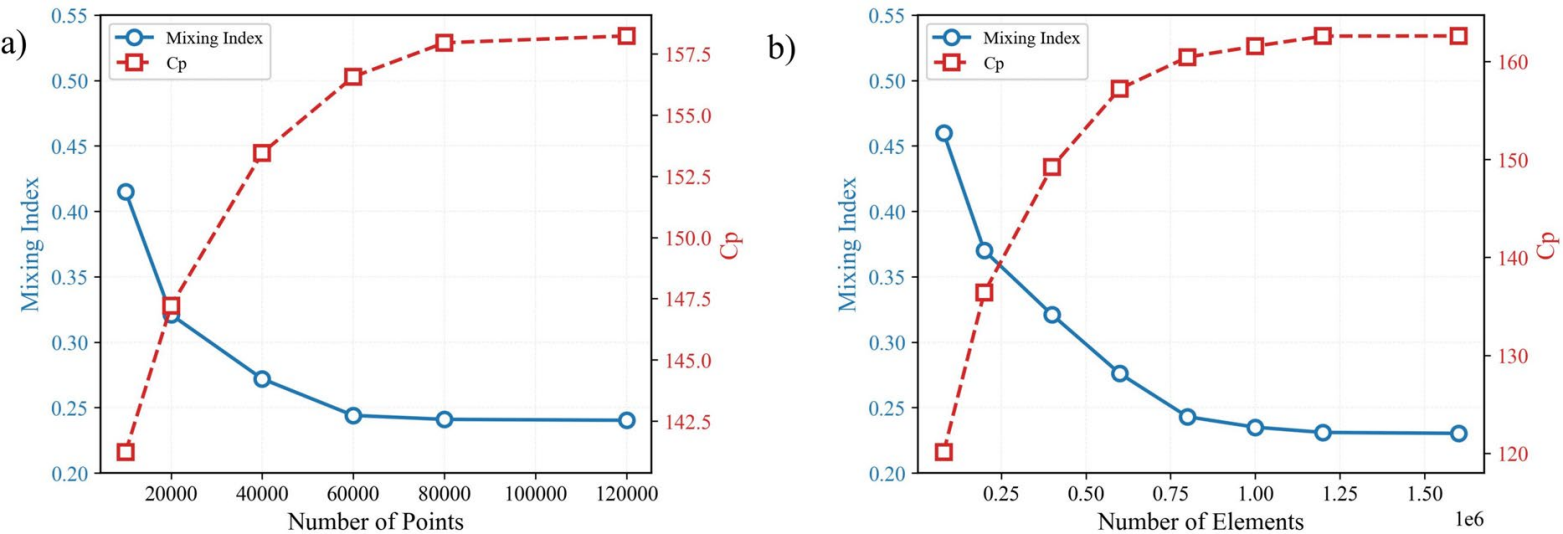
Results of the grid independence study for Configuration A (rectangular fin, Re = 5, Sc = 1000): (**a**) Convergence of the FlexPINN solution with respect to the number of collocation points. (**b**) Convergence of the reference CFD solution with respect to mesh element count.

In this section, to certify the reliability of the solution obtained using the proposed method, grid independence studies were conducted. First, as illustrated in Fig. 7a, the independence of the FlexPINN solution from the number of points was examined. Second, for comparative justification against CFD results and to ensure the reliability of the CFD solutions used throughout the study, a conventional mesh independence analysis was performed, shown in Fig. 7b. These studies were carried out for a single rectangular baffle at Reynolds number 5, Schmidt number 1000, and Configuration A. Two key performance parameters were monitored for convergence^17^. The Mixing Index (MI), defined in Eq. (38), quantifies the homogeneity of the mixture at a channel cross-section, where a value of 1 indicates perfect mixing. The Pressure Drop Coefficient (${C}_{p}$), defined in Eq. (37), represents the flow resistance normalized by the dynamic pressure. As evident from both Fig. 7a and b, further increasing the number of points in FlexPINN or refining the CFD mesh does not lead to significant changes in the predicted mixing index or pressure drop coefficient, confirming the independence of the results from these numerical discretization parameters. As shown in the figure, convergence in the results is observed upon reaching approximately 80,000 collocation points and 1,200,000 mesh elements.

39 $${C}_{p}=\frac{\Delta P}{\frac{1}{2}\rho {U}_{m}^{2}}$$

40 $$\mathrm{MI}=1-\sqrt{\frac{1}{N}\sum_{i=1}^{N}{\left(\frac{{c}_{i}^{*}-{c}_{mean}^{*}}{{c}_{mean}^{*}}\right)}^{2}}$$

### Verification

**Fig. 8:**
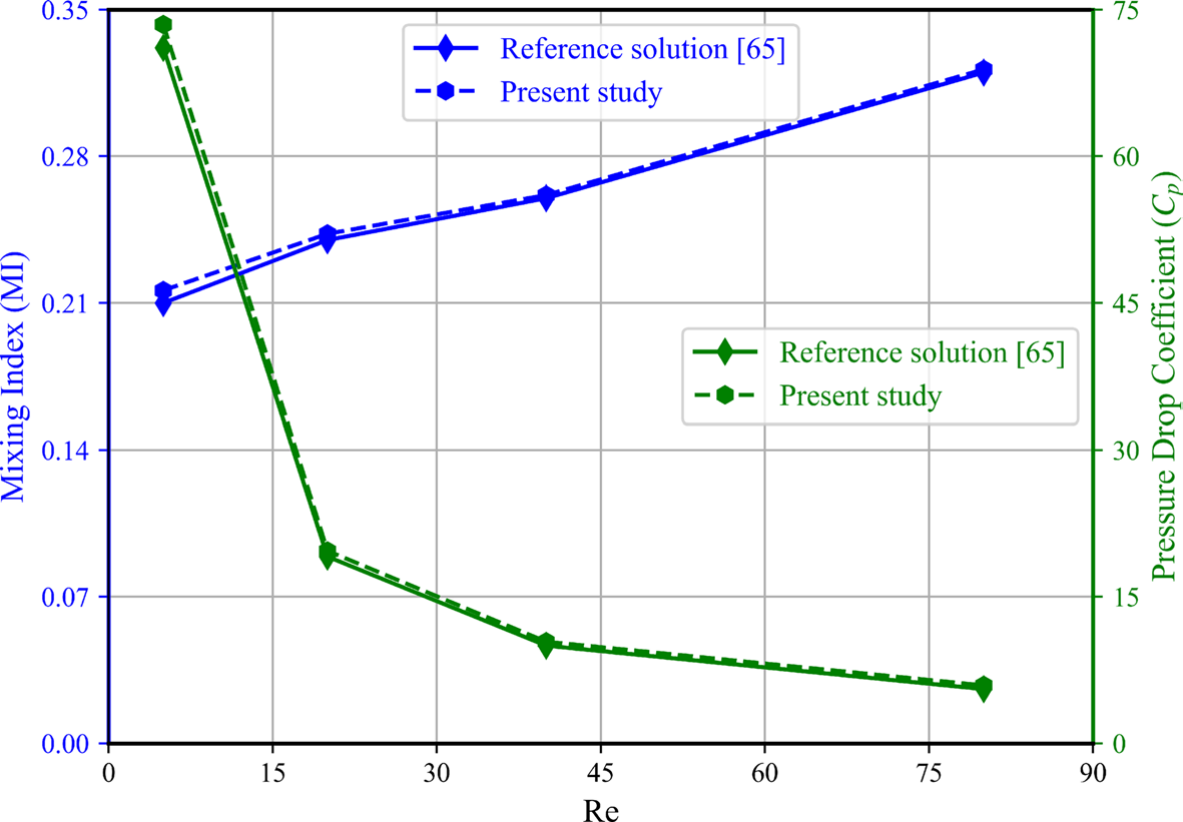
Comparison of FlexPINN results (Mixing index and pressure drop coefficient) for a simple T-mixer (no baffles) with those obtained in the study by Al-Zoubi et al.^64^ in the 3D microchannel without baffles at Sc = 1000.

To verify the results obtained from the FlexPINN method, a comparison is made with the results of similar studies. As shown in Fig. 8, the T-shape micromixer without baffles is compared with the results from Al-Zoubi et al.^64^. Two metrics, the mixing index (Eq. 37) and pressure drop coefficient (Eq. 36), are used for comparison. As shown in the figure, at low Reynolds numbers, the FlexPINN results slightly deviate from the results of Al-Zoubi et al. However, the maximum relative error with respect to their study is reported as 2.86% in the mixing index and 3.25% in the pressure drop coefficient.

**Fig. 9:**
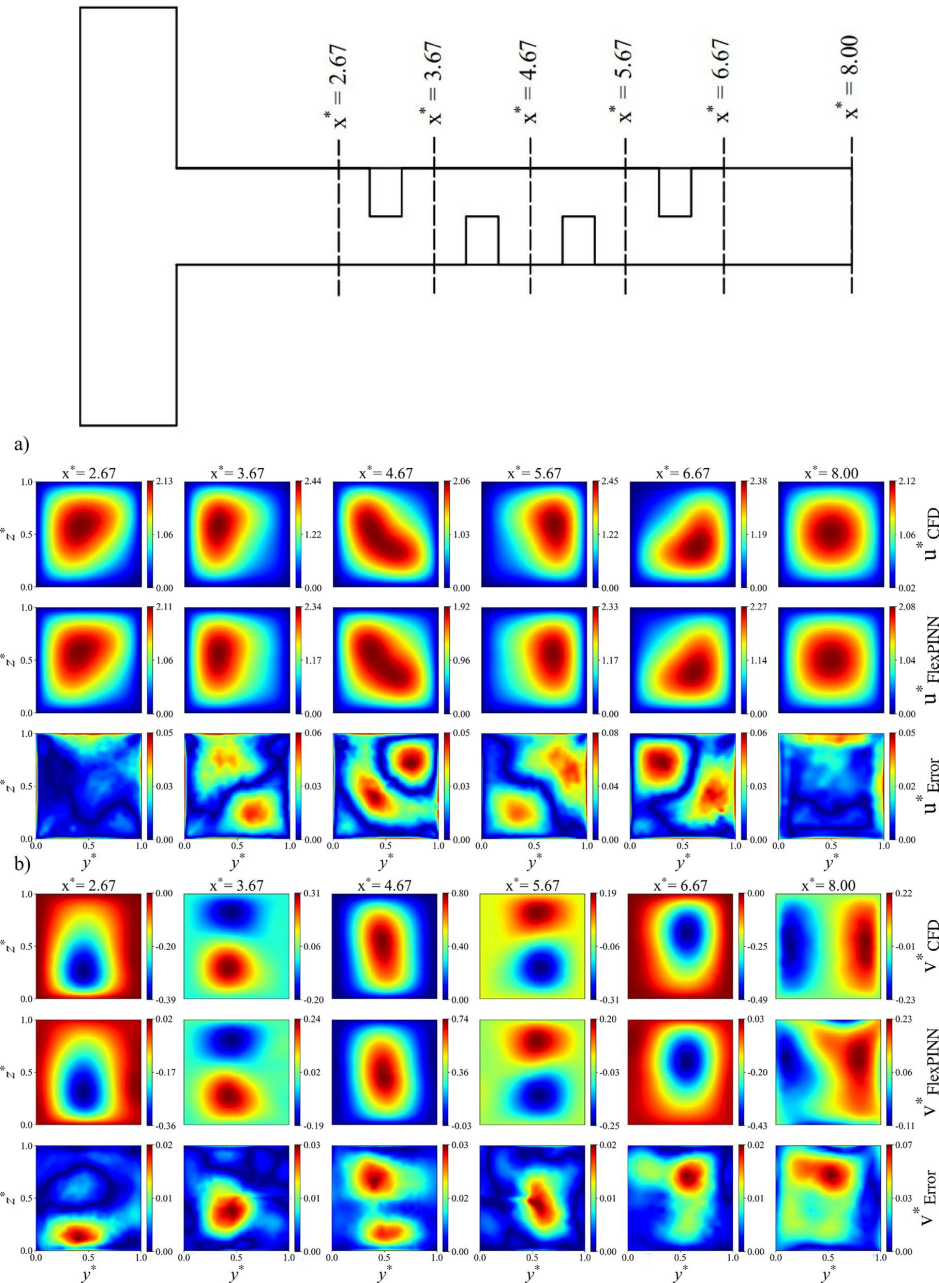

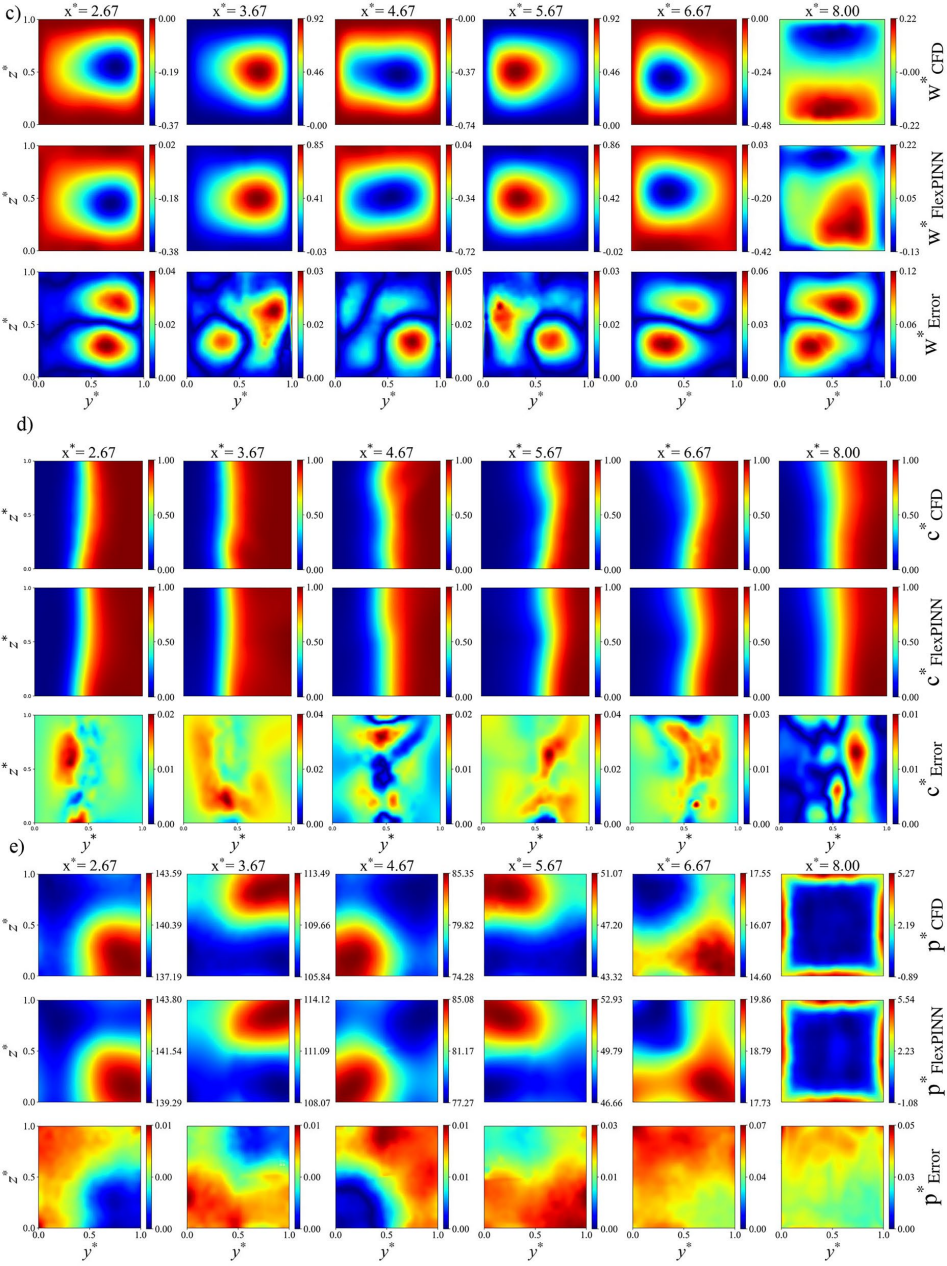
Comparison of the results obtained from the FlexPINN method with CFD results at various cross-sections of the microchannel in configuration A, Re = 5, Sc = 1000, rectangular shape, and single-unit: (**a**) dimensionless velocity in x direction, (**b**) dimensionless velocity in y direction, (**c**) dimensionless velocity in z direction, (**d**) dimensionless concentration, and (**e**) dimensionless pressure.

To further validate the results obtained using the FlexPINN method, the single-unit configuration at Re = 5 was compared against conventional CFD solutions. Figure 9 presents contour plots of the dimensionless velocity components, concentration, and pressure for a microchannel with rectangular baffles in Configuration A, shown at various cross-sections along the channel. As illustrated, the FlexPINN predictions closely match the CFD results, demonstrating high accuracy. The maximum error in the dimensionless horizontal velocity is reported to be 0.08, which is negligible compared to the inlet dimensionless concentration value of unity. Additional results for other configurations can be found at the https://github.com/imRaajee/FlexPINN.

**Fig. 10:**
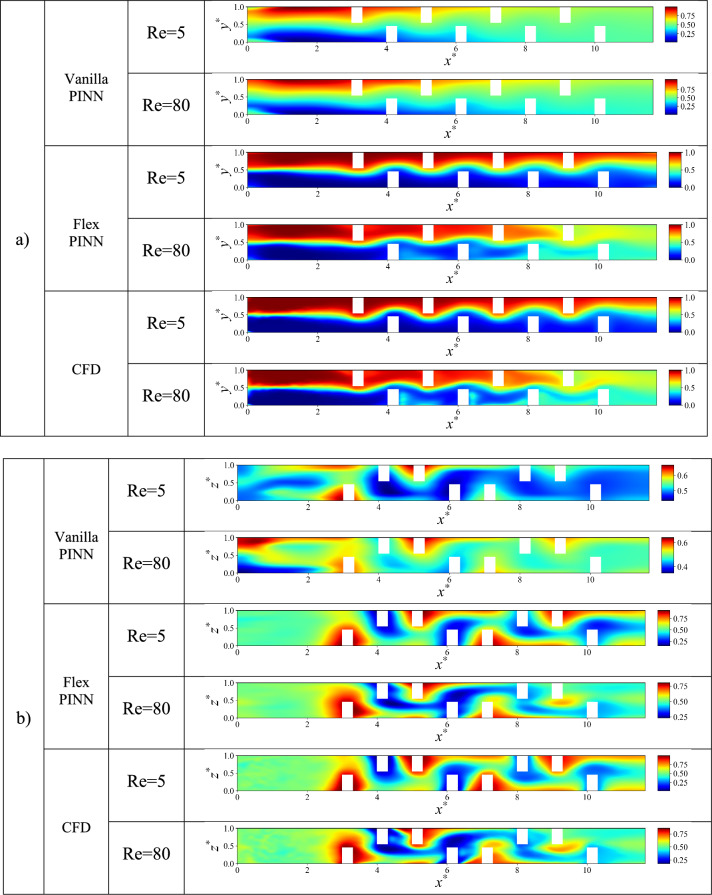
Comparison of dimensionless concentration contours between vanilla PINN, FlexPINN, and CFD methods in Re = 5 and Re = 80 (Sc = 1000). (**a**) mid $xy$-plane, (**b**) mid $xz$-plane.

It is worth mentioning that the vanilla PINN was first implemented to simulate the problem. However, due to the three-dimensional nature of the baffles inside the channel, the vanilla PINN fails to accurately predict the concentration distribution. To solve this issue, FlexPINN wes introduced to simulate the complicated fluid mechanics problems. Figure 10 compares the dimensionless concentration contours obtained using the vanilla PINN and the FlexPINN method for two different Reynolds numbers in the double-unit channel. The CFD results is shown in this figure for the same problem for comparing vanilla PINN and FlexPINN methods. As shown in the figure, the FlexPINN method provides an accurate representation of the governing fluid dynamics.

### Parametric study

#### Effect of reynolds number and baffle configurations

**Fig. 11:**
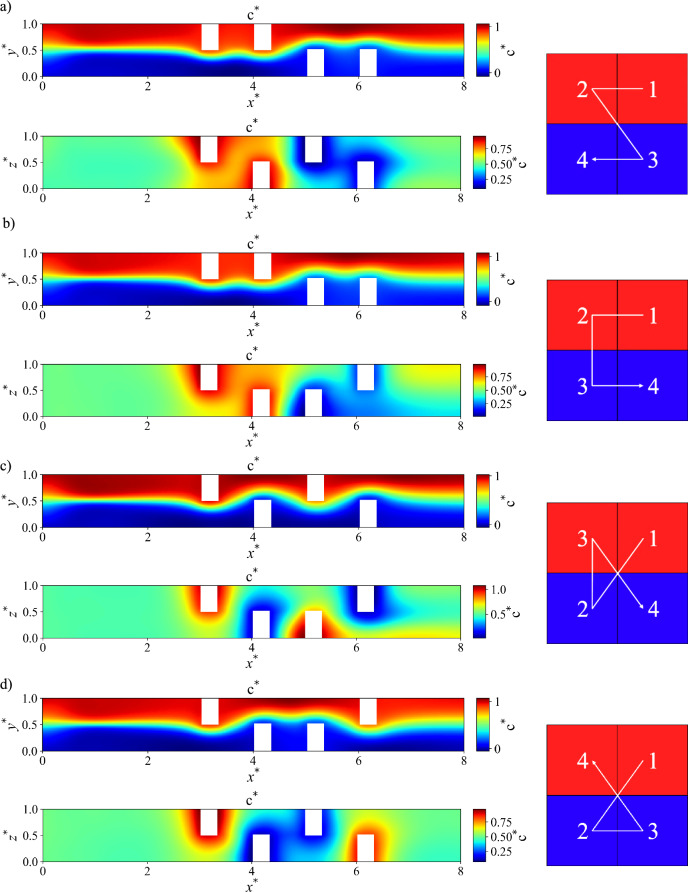
Contours of dimensionless concentration for baffle configurations in the mid $xy$-plane (top), mid $xz$-plane (bottom), and flow direction (right) in a single-unit configuration with rectangular baffles at Re = 5 and Sc = 1000. (**a**) Configuration A, (**b**) Configuration B, (**c**) Configuration C, and (**d**) Configuration D.

The effect of different configurations is examined in Fig. 11, where the contour of dimensionless concentration for various configurations in a single-unit channel with rectangular baffles is analyzed. These contours are displayed at the middle section of the microchannel in both the $xy$ and $xz$ planes. Contour plots for all cases, including dimensionless velocity components and dimensionless pressure for different configurations, are provided on the GitHub repository. As illustrated in the figures, the baffles are strategically arranged to guide the flow along the directions indicated by the arrows. Each configuration features a distinct baffle arrangement within the microchannel, which alters the flow path accordingly. The interaction between the two fluid streams is visually represented by the intersection of arrows with the interface between the blue and red regions. In Fig. 11a and b, the arrows intersect the interface only once, indicating a single oscillation and limited mixing. In contrast, Fig. 11c and d exhibit more complex flow paths, where the arrows cross the interface three and two times, respectively, suggesting enhanced mixing due to increased fluid interaction. While this figure qualitatively illustrates the influence of baffle configuration on flow behavior and mixing dynamics, a quantitative comparison of these four configurations is provided in the subsequent analysis.

**Fig. 12:**
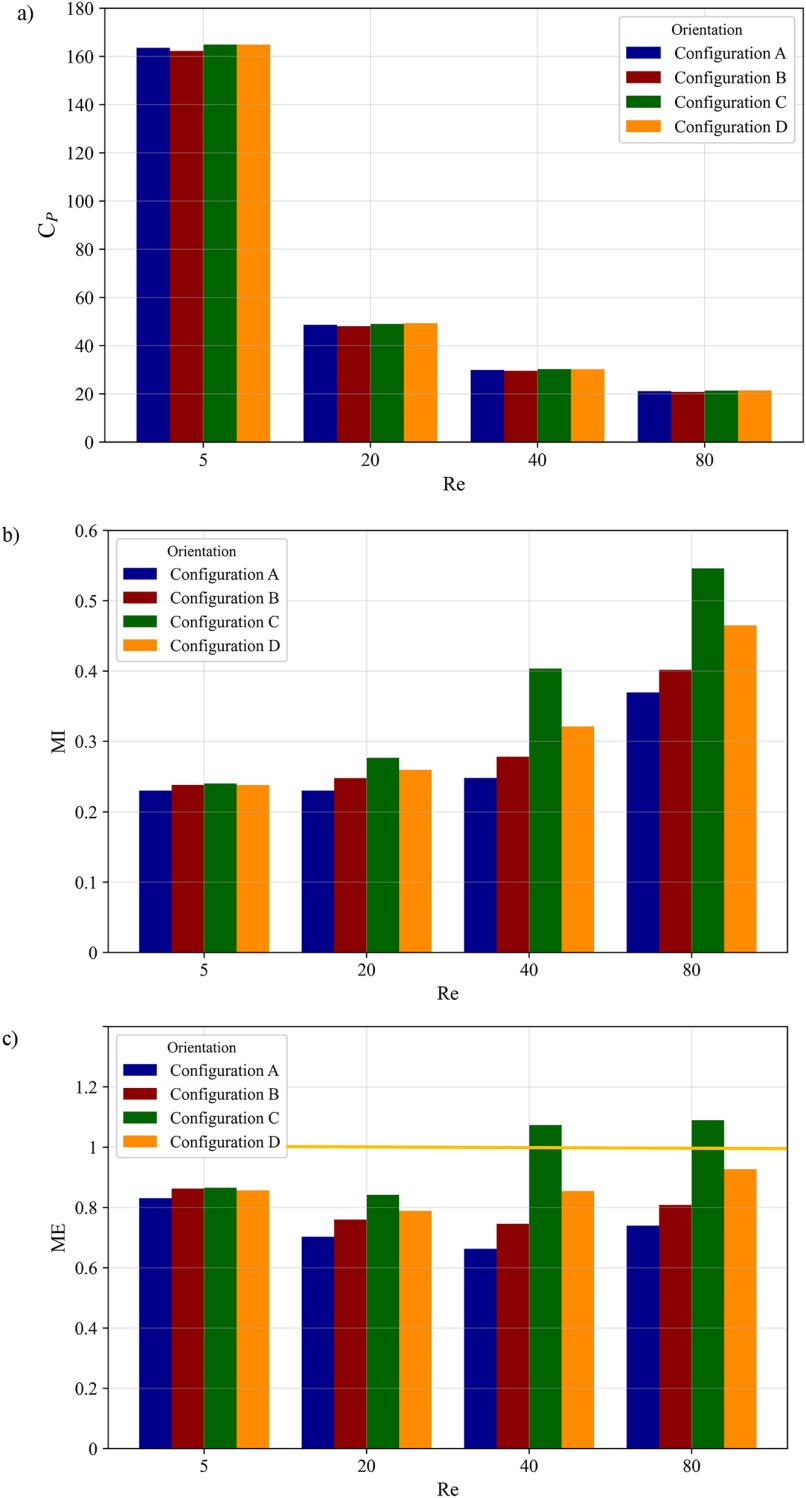
Effect of baffle configuration and Reynolds number on: (**a**) pressure drop coefficient, (**b**) mixing index, and (**c**) mixing efficiency in a single-unit microchannel at Sc = 1000.

To comprehensively evaluate the impact of baffle configurations on micromixing performance, Fig. 12 presents the variations in pressure drop coefficient, mixing index, and Mixing Efficiency (ME) (as defined by Eq. 42^20^) across different baffle configurations and Reynolds numbers. The comprehensive ME, evaluates the trade-off by comparing the improvement in MI relative to the incurred ${C}_{p}$ where mixing enhancement justifies the added pumping cost.

41 $$\mathrm{ME}=\frac{\frac{\mathrm{MI}}{{\mathrm{MI}}_{0}}}{{\left(\frac{{C}_{p}}{{C}_{p,0}}\right)}^\frac{1}{3}}$$

In this equation, the subscript zero denotes the reference case (without baffle), which was used for initial validation purposes. Figure 12(a) shows that the pressure drop coefficient (${C}_{p}$) decreases with increasing Reynolds number for all configurations, a trend driven by inertial dominance. At higher Re, the reduced relative influence of viscosity leads to thinner boundary layers and a more streamlined core flow, lowering the normalized pressure loss. For example, ${C}_{p}$ for Configuration A decreases by approximately 87.5% between Re = 5 and Re = 80. Notably, the specific baffle configuration (Configurations A–D) has a minor influence on ${C}_{p}$ (variation < 2% at a given Re), indicating that the pressure penalty in these designs is governed more by the presence of the baffles themselves than by their specific sequential placement.

Figure 12b demonstrates a consistent increase in MI with Reynolds number across all configurations, with Configuration C achieving the highest performance (MI ≈ 0.545 at Re = 80, ~ 33% higher than Configuration A at the same Re). This superior performance stems from its irregular, staggered baffle arrangement, which maximizes streamline crossing and induces chaotic advection within the laminar regime. The staggered layout continuously redirects the fluid streams, stretching and folding the interface to enhance diffusive flux. Configuration D, with a moderate number of interface crossings, follows in performance. In contrast, the more symmetrical arrangements of Configurations A and B produce less flow disruption, resulting in lower MI values. When assessing the trade-off with energy cost via the ME metric (Eq. 42), Configuration C again excels, achieving ME > 1 across the Re range. This confirms that its significant mixing enhancement (e.g., ~ 37.5% increase in MI over the baseline at Re = 40) justifies the incurred pressure penalty, establishing it as the most efficient design for the conditions studied and justifying its selection for further analysis. Furthermore, in Fig. 12c, a mixing efficiency of 1, which indicates optimal mixing performance for the baffled configuration compared to the case without a baffle, is highlighted by a yellow line.

#### Effect of the baffle shape in a single-unit microchannel

**Fig. 13:**
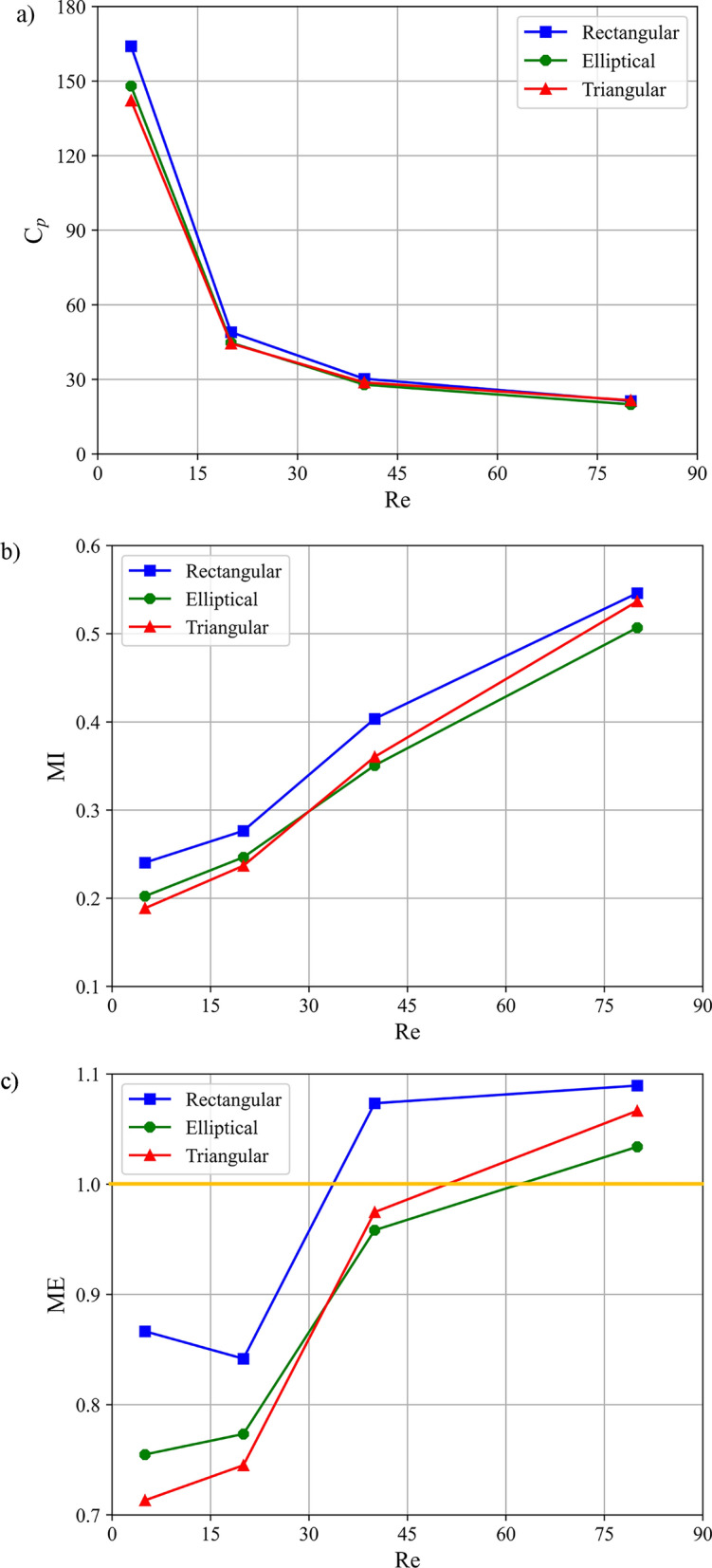
Influence of Reynolds number and baffle shape on: (**a**) pressure drop coefficient, (**b**) mixing index at the channel outlet, and (**c**) mixing efficiency in single unit microchannel at Sc = 1000.

Based on its optimal mixing efficiency, Configuration C was selected for a detailed analysis of baffle shape. Figure 13 quantifies the effect of baffle shape (rectangular, elliptical, triangular) and Reynolds number on performance. Consistent with prior observations, increasing Re reduces the ${C}_{p}$ and increases both the MI and ME for all shapes. The rectangular baffle consistently yields the highest values across all metrics; for instance, at Re = 80, it achieves an MI of ~ 0.56 and a ${C}_{p}$ of ~ 19, resulting in an ME of 1.09, approximately 5.8% higher than the elliptical baffle and 2.8% higher than the triangular baffle. This superior performance is directly attributable to its sharp-edged geometry, which generates the strongest flow separation and recirculation zones, thereby maximizing interfacial stretching and chaotic advection. A notable transition in ME trends occurs near Re = 20 for all shapes (Fig. 12c), indicating a shift in the dominant mixing mechanism. This threshold is analyzed in the context of established mixing regimes in the following discussion.

The performance trends can be interpreted through the lens of established mixing regimes: diffusive (Re <  ~ 10), transient (~ 10 < Re <  ~ 50), and chaotic (Re >  ~ 50)^15^. The data in Fig. 13c reflects these transitions. For rectangular baffles, ME shows a non-monotonic dip near Re = 20 before rising sharply. In the diffusive-dominated regime (Re < 10), the significant pressure penalty (${C}_{p}$~ 165 at Re = 5) imposed by its sharp edges outweighs the limited convective mixing gain, reducing ME. As inertia increases (Re > 20), the strong, chaotic vortices generated by these same edges become highly effective, leading to a rapid ME increase—reaching 1.075 at Re = 40. In contrast, the streamlined profiles of elliptical and triangular baffles impose a lower initial pressure penalty (${C}_{p}$~ 148 and ~ 143 at Re = 5, respectively), resulting in a steadier ME increase with Re. Below Re = 30, elliptical baffles outperform triangular ones (e.g., ~ 7% higher ME at Re = 5), as their curved surface promotes smoother, yet sustained, fluid redirection and interfacial stretching conducive to laminar enhancement. At higher Re (chaotic regime), the flow-separating sharp edges of the triangular baffle become more advantageous, narrowing the performance gap (e.g., ~ 3.8% lower ME at Re = 80). In summary, the choice of baffle shape presents a clear trade-off: rectangular baffles maximize ultimate mixing performance and efficiency at moderate-to-high Re by aggressively disrupting flow, elliptical baffles offer an efficient balance for low-Re applications by enhancing mixing with minimal pressure cost, and triangular baffles provide an intermediate strategy, with performance improving in chaotic flows. A mixing efficiency of unity, representing the optimal mixing performance achieved with the baffle relative to the unbaffled case, is delineated by a yellow line in Fig. 13c.

#### Effects of the baffle shape in a double-unit microchannel

**Fig. 14:**
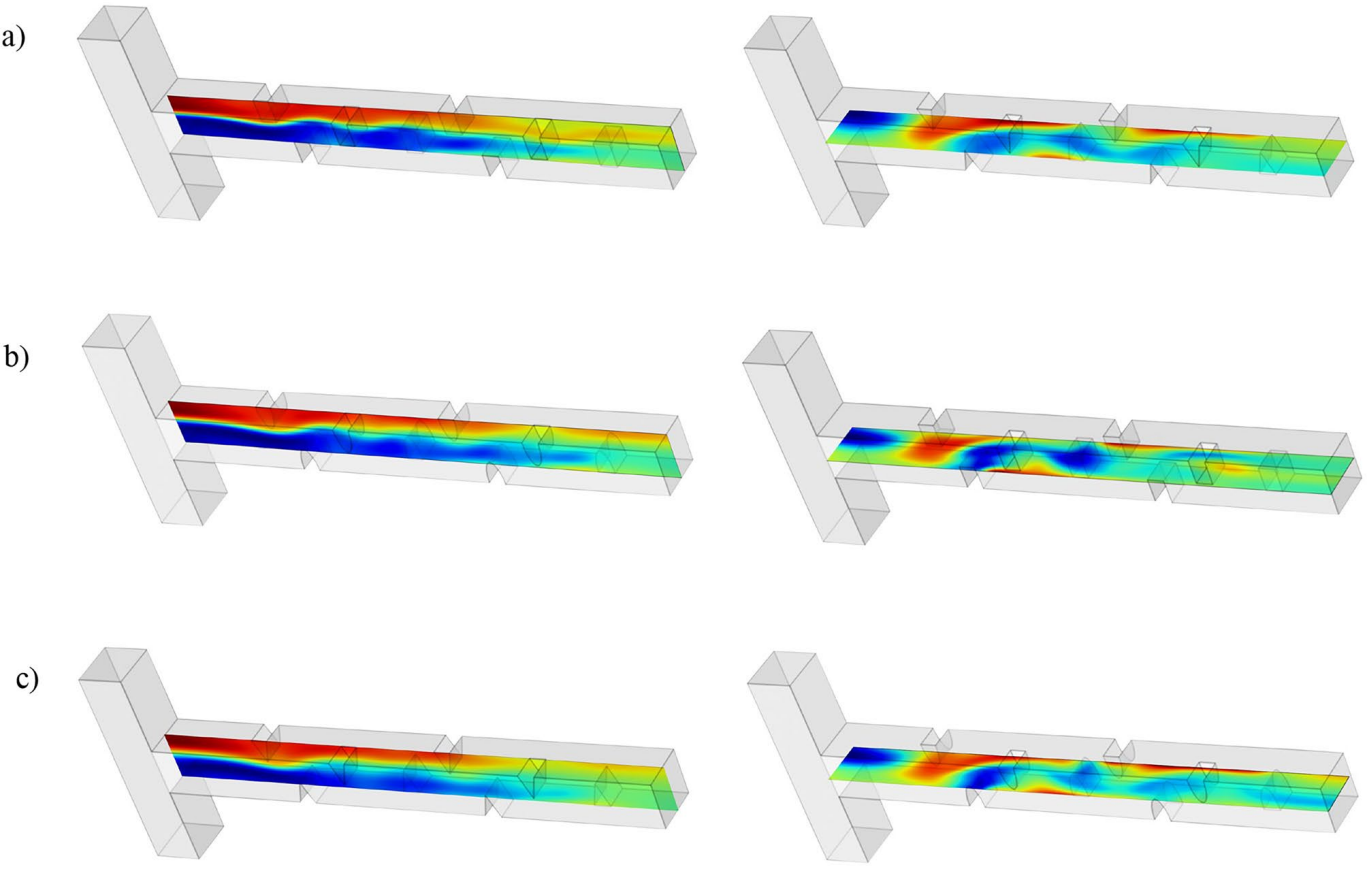
Dimensionless concentration contours in the $\mathrm{xy}$-plane (left) and $\mathrm{xz}$-plane (right): (**a**) rectangular baffles, (**b**) elliptical baffles, and (**c**) triangular baffles in a double-unit configuration C at Reynolds number Re = 80 and Sc = 1000.

In this section, the performance of the double-unit microchannel configuration is further assessed. Figure 14 presents dimensionless concentration contours in both the xz-plane (horizontal cross-section) and xy-plane (vertical cross-section) for the three distinct baffle shapes arranged in Configuration C at a Reynolds number of 80. The contours visually depict how each baffle geometry influences the distribution and mixing of the two fluid streams. These contour plots provide a qualitative insight into the mixing mechanisms enhanced by the extended channel length and baffle configuration, setting the stage for subsequent quantitative analysis of mixing efficiency and pressure drop characteristics.

**Fig. 15:**
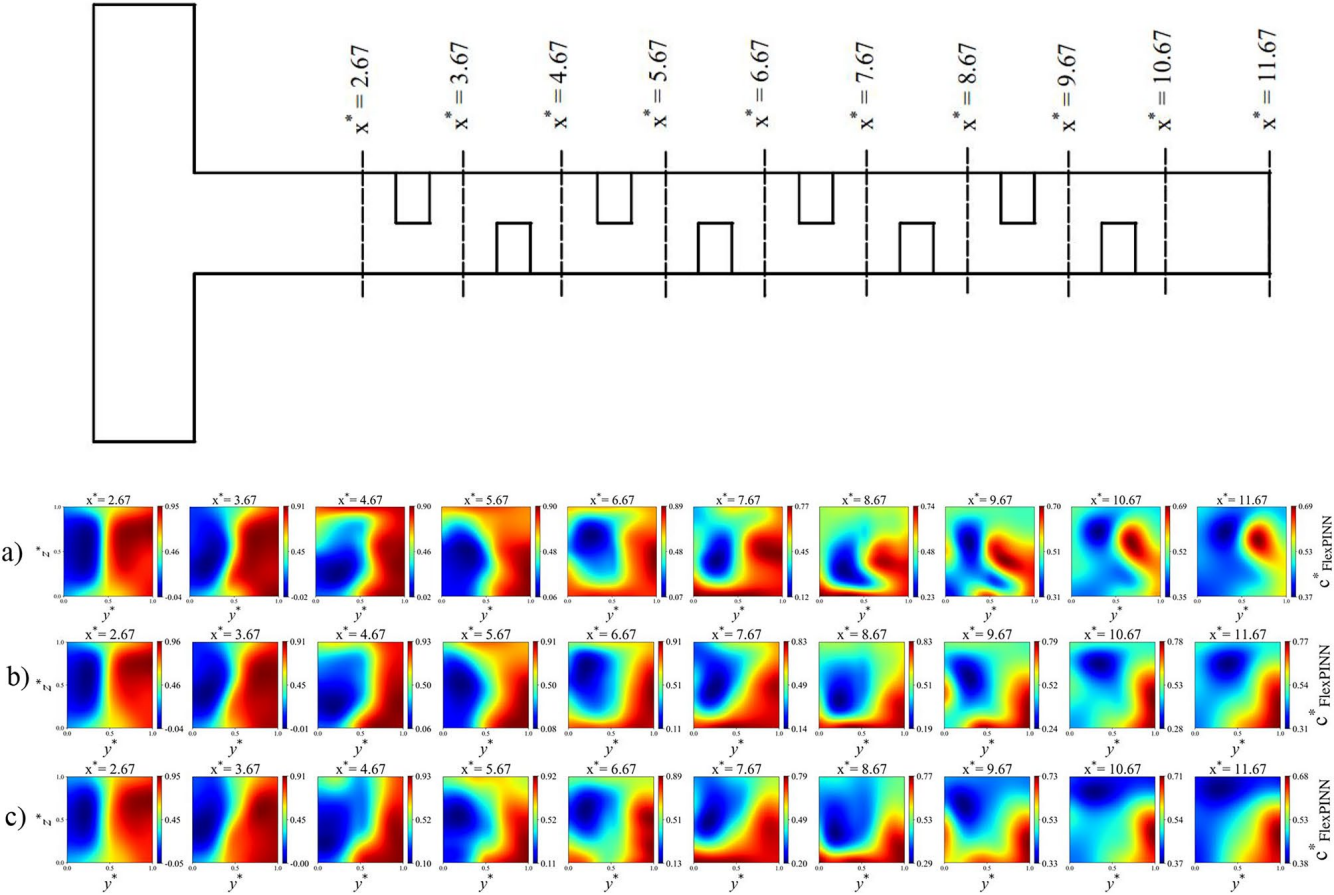
Dimensionless concentration contours along the microchannel at Reynolds number 80 and Sc = 1000: (**a**) Rectangular baffles, (**b**) Elliptical baffles, (**c**) Triangular baffles.

Figure 15 further extends the qualitative analysis by displaying the evolution of dimensionless concentration contours along the entire length of the microchannel at Reynolds number 80 for all three baffle shapes. The sequential contour plots reveal how mixing develops progressively from the inlet to the outlet. Additional results, including dimensionless velocity and pressure contours along the microchannel, are available at the GitHub link. As observed, the rectangular baffles exhibit superior mixing performance compared to the elliptical and triangular baffles. This section provides a qualitative assessment, while a more detailed quantitative analysis and explanation will be presented in the subsequent sections.

**Fig. 16:**
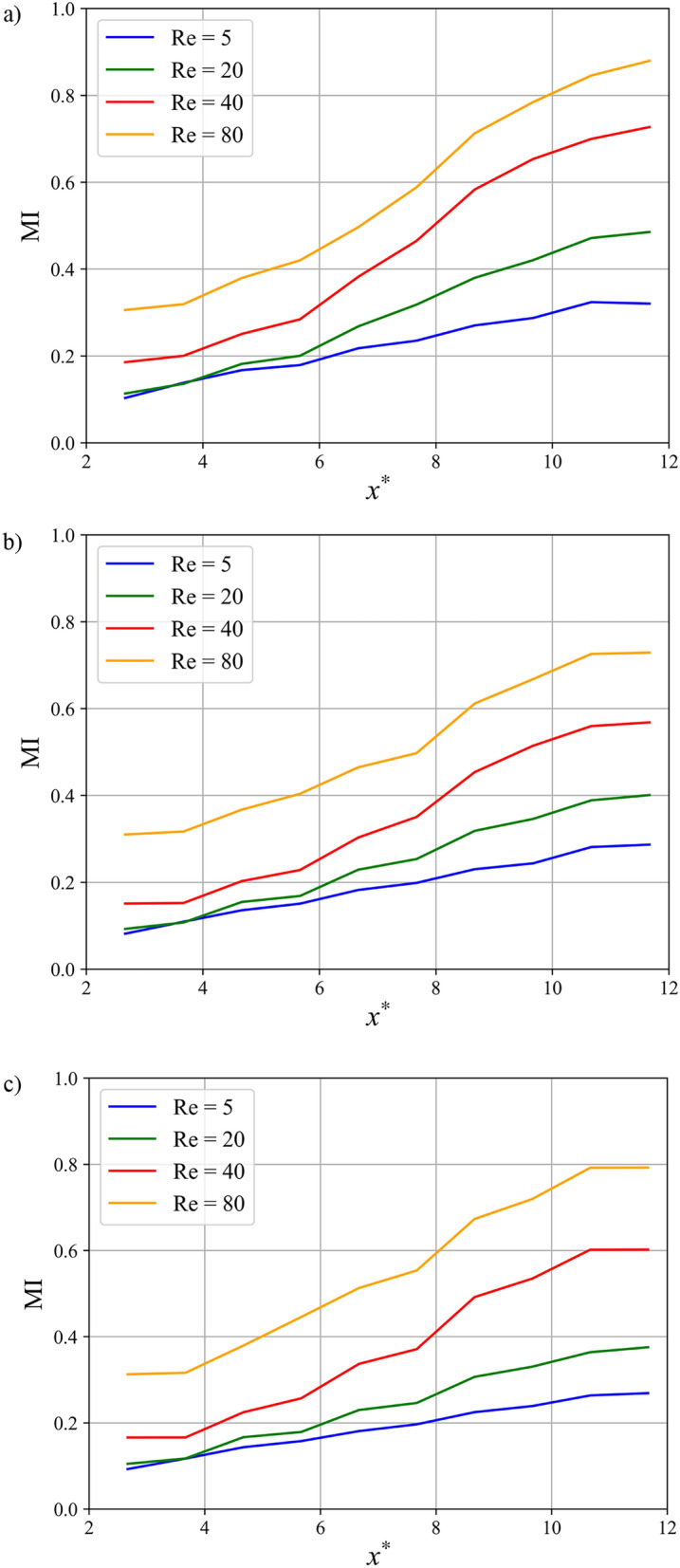
Effect of Reynolds number on the mixing index along the microchannel at Sc = 1000: (**a**) rectangular baffles, (**b**) elliptical baffles, and (**c**) triangular baffles in double unit configuration C.

Figure 16 quantifies the evolution of the MI along the channel length for all baffle shapes. As expected, MI increases with both Reynolds number and microchannel length. At the outlet, the rectangular baffle achieves the highest MI, reaching ~ 0.88 at Re = 80, compared to ~ 0.72 for the elliptical and ~ 0.79 for the triangular baffles at the same condition. The more pronounced longitudinal MI gradient at low Re (e.g., Re = 5) underscores the dominant role of extended residence time for molecular diffusion. In contrast, at high Re (Re = 80), the MI rises more steeply in the initial baffled sections before plateauing, indicating that chaotic advection induced by the baffles rapidly achieves mixing, diminishing the relative benefit of additional channel length. The most significant MI improvements occur in the transitional Re range (20–40), where inertia begins to effectively complement diffusion, highlighting the shift from a diffusion-dominated to a convection-dominated mixing mechanism.

**Fig. 17:**
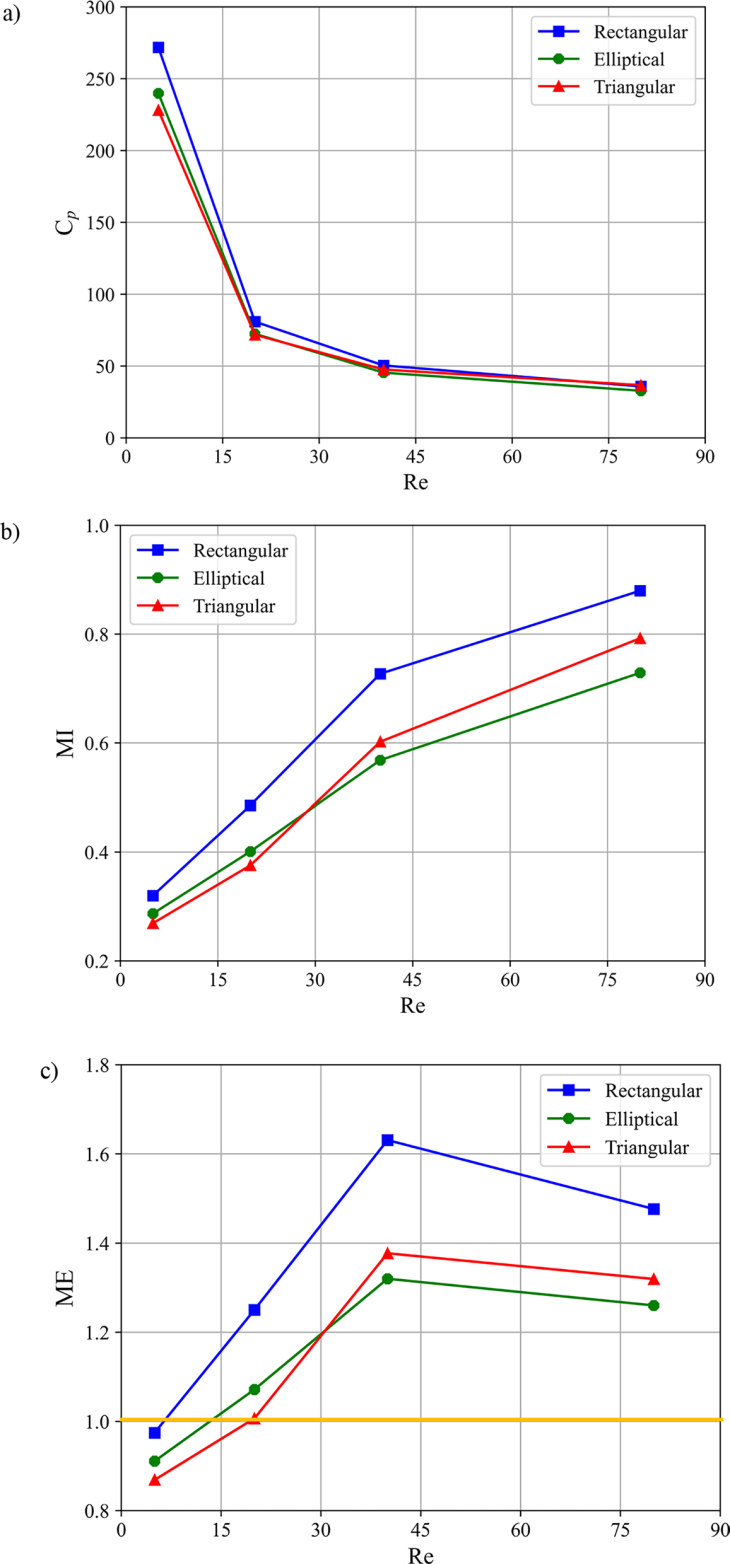
Effect of Reynolds number and baffle shape in the double-unit microchannel configuration C and Sc = 1000 on: (**a**) pressure drop coefficient, (**b**) mixing index at outlet of microchannel, and (**c**) mixing efficiency.

Figure 17 summarizes the performance of the double-unit micromixer. Consistent with single-unit results, the MI increases with Reynolds number for all baffle shapes, while the ${C}_{p}$ decreases. However, the extended channel length amplifies the pressure penalty at higher flow rates. Consequently, the ME peaks for all shapes at Re ~ 40 (e.g., ME ≈ 1.63 for rectangular baffles) and declines at Re = 80. This decline occurs because the cumulative pressure loss along the longer channel increases more rapidly with Re (approximately quadratically) than the accompanying gain in MI, reducing the benefit-to-cost ratio. Rectangular baffles maintain the highest ME across the Re range, outperforming elliptical baffles by ~ 18% and triangular baffles by ~ 23% at Re = 40. A distinct performance crossover is observed around Re = 30. Elliptical baffles dominate at lower Reynolds numbers (e.g., with a ~ 3.4% higher ME at Re = 5), where their streamlined shape is advantageous. Above this threshold, the flow regime shifts, and the geometry of triangular baffles prevails, yielding superior performance (e.g., a ~ 3.9% higher ME at Re = 80). In summary, the double-unit results reinforce that ME is a balance between achieving chaotic advection/interfacial stretching (high MI) and minimizing resistive dissipation (low ${C}_{p}$). The extended channel length intensifies the pressure penalty, shifting the optimal efficiency peak to a moderate Re (~ 40) and altering the comparative advantages of each baffle shape across the Reynolds number spectrum.

In this study, the FlexPINN framework has successfully simulated fluid flow and mass transfer phenomena in a 3D microchannel equipped with baffles. This model can be further extended through parametric studies to explore the full potential of the method. Moreover, incorporating data from other numerical approaches for use in transfer learning^65^, or integrating with alternative neural network structures^66^, may offer valuable avenues for enhancing the capabilities of FlexPINN.

## Conclusion

This study successfully bridges a critical gap in both micromixer design methodology and computational simulation by introducing and validating the Flexible Physics-Informed Neural Network (FlexPINN) for complex three-dimensional flows. Unlike prior CFD-based investigations of chaotic 3D mixers^27^ or curved channels with baffles^30^, which rely on computationally intensive meshing, FlexPINN provides a mesh-free, physics-informed framework capable of efficiently simulating intricate, baffle-laden geometries. The framework’s key innovations enabled the robust solution of a previously intractable problem for standard PINNs: fully 3D laminar flow and mass transfer in a straight microchannel with an array of internal baffles.

Our comprehensive parametric study yields concrete, quantitative design insights. We demonstrate that within a straight-channel architecture, the specific corner-wise configuration of baffles is a primary performance driver. The optimal design (rectangular baffles in a staggered double-unit arrangement (Configuration C) at Re = 40) achieved a ME of 1.63, indicating a favorable balance where the gain in mixing index significantly outweighs the associated pressure penalty. This configuration outperformed elliptical and triangular shapes across most Reynolds numbers, with rectangular baffles yielding the highest mixing index (up to ~ 0.88 at the outlet for the double-unit case at Re = 80). Notably, the proposed method achieved high predictive accuracy, with maximum relative errors of 3.25% for the pressure drop coefficient and 2.86% for the mixing index compared to reference CFD data, while transfer learning reduced training time for new baffle shapes by approximately 35%. These results advance the field in two key ways. First, they provide a validated computational tool (FlexPINN) that circumvents the meshing bottleneck for 3D parametric studies, offering a new pathway for rapid microfluidic device exploration. Second, they deliver novel, generalizable design principles for passive straight-channel micromixers, showing that strategic baffle sequencing can induce effective 3D flow twisting and chaotic advection without requiring channel curvature, thus simplifying fabrication. Designers can use the provided performance maps (e.g., Figs. 12, 13, 17) to select a baffle shape and arrangement that meets specific mixing index (MI) and mixing efficiency (ME) targets for their application.

Future work should focus on several promising directions: (1) Experimental validation and manufacturing of the optimal configurations identified here to confirm their practical performance. (2) Integration of FlexPINN into a full parametric optimization loop, where geometric parameters become direct network inputs, enabling automated optimal design. (3) Extension of the framework to model transitional flows, conjugate heat transfer, or reactive mixing to tackle more complex multi-physics applications. (4) Exploration of hybrid data-physics approaches, incorporating sparse experimental data to further enhance predictive reliability for novel geometries.

## Data Availability

The python codes used to generate the results presented are available on Github. https://github.com/imRaajee/FlexPINN.

## Dimensionless variables

${\tau }_{xx}^{*},{\tau }_{yy}^{*},{\tau }_{zz}^{*}$: Dimensionless normal stress components (–)
${\tau }_{xy}^{*},{\tau }_{xz}^{*},{\tau }_{yz}^{*}$: Dimensionless shear stress components (–)
${J}_{x}^{*},{J}_{y}^{*},{J}_{z}^{*}$: Dimensionless concentration flux components (–)
$${x}^{*}$$: Dimensionless *x*-coordinate (–)
$${y}^{*}$$: Dimensionless *y*-coordinate (–)
$${z}^{*}$$: Dimensionless *z*-coordinate (–)

## Physical variables

$u,v,w$: Velocity components in *x*,*y*,*z* directionsm (s^-1^)
$$\overrightarrow{U}$$: Velocity vectorm (s^-1^)
$$p$$: Pressure (Pa)
$$c$$: Concentration (mol m^-3^)

## Geometric parameters

$L \left(1 \,Unit\right)$: Length of single-unit microchannel (mm)
$L \left(2 \,Units\right)$: Length of double-unit microchannel (mm)
${L}_{0}$: First baffle inlet distance (mm)
${L}_{1}$: Length of the inlet section (mm)
$D$: Depth of the microchannel (mm)
$H$: Height of the microchannel (mm)
$W$: Width of the microchannel inlet (mm)
$d$: Depth of the baffle (mm)
$h$: Height of the baffle (mm)
$w$: Width of the baffle (mm)
*l*: Distance between two consecutive baffles (mm)

## Fluid properties & parameters

$$\rho$$: Fluid densitykg (m^-3^)
$\mu$: Dynamic viscosity (Pa s)
$${D}_{c}$$: Diffusion coefficient (m^2^ s^-1^)
${U}_{mean}$: Mean velocity in the main channelm (s^-1^)

## Dimensionless numbers

$$\mathrm{Re}$$: Reynolds number (–)
$$\mathrm{Sc}$$: Schmidt number

## Performance metrics

$$\mathrm{MI}$$: Mixing index (–)
$Cp$: Pressure drop coefficient (–)
$$\mathrm{ME}$$: Mixing efficiency (–)
$\Delta P$: Pressure drop across the microchannel (Pa)
$N$: Number of sampling points

## Mathematical operators & misc

$$\nabla$$: Gradient operator (–)
$${\nabla }^{2}$$: Laplacian operator (–)
$$\frac{\partial }{\partial x},\frac{\partial }{\partial y},\frac{\partial }{\partial z}$$: Partial derivatives (–)
${n}_{x},{n}_{y},{n}_{z}$: Components of the unit normal vector (–)
$\theta$: Neural network parameters (weights & biases) (–)
${L}_{GE}$: Loss term for governing equations (–)
${L}_{BC}$: Loss term for boundary conditions (–)
${L}_{PT}$: Loss term for penalty terms (–)
${L}_{Tot}$: Total loss function (–)

## Subscripts

$0$: Reference case (without baffles) (–)
$i$: Index for sampling points (–)
inlet: Inlet of the channel
$wall$: Evaluated at the wall (–)
$$mean$$: Mean value (–)
$*$: Denotes dimensionless quantity
